# Thioquinoline derivatives conjugated to thiosemicarbazide as potent tyrosinase inhibitors with anti-melanogenesis properties

**DOI:** 10.1038/s41598-023-28852-1

**Published:** 2023-02-13

**Authors:** Milad Noori, Reyhaneh Sabourian, Ali Tasharoie, Maliheh Safavi, Aida Iraji, Minoo Khalili Ghomi, Navid Dastyafteh, Cambyz Irajie, Elham Zarenezhad, Seyyed Mehrdad Mostafavi Pour, Fatemeh Rasekh, Bagher Larijani, Mohsen Amini, Mannan Hajimahmoodi, Mohammad Mahdavi

**Affiliations:** 1 Endocrinology and Metabolism Research Center, Endocrinology and Metabolism Clinical Sciences Institute, Tehran University of Medical Sciences, Tehran, Iran; 2 Drug and Food Control Department, Faculty of Pharmacy, Tehran University of Medical Sciences, Tehran, Iran; 3 Department of Biotechnology, Iranian Research Organization for Science and Technology (IROST), Tehran, Iran; 4 Stem Cells Technology Research Center, Shiraz University of Medical Sciences, Shiraz, Iran; 5 Central Research Laboratory, Shiraz University of Medical Sciences, Shiraz, Iran; 6 Department of Medical Biotechnology, School of Advanced Medical Sciences and Technologies, Shiraz University of Medical Sciences, Shiraz, Iran; 7 Liosa Pharmed Parseh Company, Shiraz, Iran; 8 Department of Biology, Payame Noor University(PNU), Tehran, Iran; 9 Department of Pharmaceutical Biotechnology, Faculty of Pharmacy, Tehran University of Medical Sciences, Tehran, Iran; 10 Drug and Food Control Department, Faculty of Pharmacy, Tehran University of Medical Sciences, Tehran, Iran; 11 Halal Research Center of IRI, Food and Drug Administration, Ministry of Health and Medical Education, Tehran, Iran

**Keywords:** Chemical biology, Drug discovery

## Abstract

In the present study, a series of aryl-substituted thioqunoline conjugated to thiosemicarbazide were rationally designed and synthesized. The formation of target compounds was confirmed by spectral characterization techniques such as IR, ^1^H-NMR, ^13^C-NMR, ESI–MS, and elemental analysis. Among the synthesized derivatives, compound **10g** bearing *para*-chlorophenyl moiety was proved to be the most potent tyrosinase inhibitor with an IC_50_ value of 25.75 ± 0.19 µM. Compound **10g** as the most potent derivative exhibited a noncompetitive inhibition pattern against tyrosinase in the kinetic study. Furthermore, the in silico cavity detection, as well as the molecular docking assessments, were performed to follow the behavior of **10g** within the proposed binding site. Besides, the toxicity of **10g** and its potency to reduce the melanin content on A375 cell lines were also measured. Consequently, aryl-substituted thioqunolines conjugated to thiosemicarbazide might be a promising candidate in the cosmetics, medicine, and food industry as tyrosinase inhibitors.

## Introduction

Melanin is a group of biopolymer pigments that is a mixture of dark brown or black eumelanin and lighter-colored pheomelanin in many organisms^1^. In humans, it protects the skin from UV radiation and other environmental challenges and gives the skin it's color^2,3^. However, the excessive accumulation of melanin might result in some disorders, such as melanoma, melasma, and freckles as well as increase the risk of cancer^4,5^ and Parkinson's disease^6^. Also, the undesirable phenomena of enzymatic browning in most fruits and vegetables as well as in seafood leading to brown or black pigments on products should not be neglected. Melanogenesis is the biochemical pathway leading to the synthesis of melanin in which tyrosinase as a key enzyme hydroxylates tyrosine to L-Dopa and oxidizes it to dopaquinone, which polymerizes spontaneously to form melanin^2,7^.

Tyrosinase (EC 1.14.18.1) is a copper-containing metalloenzyme that is widely distributed in microorganisms, plants, and animals^8^. Crystal structures of mushroom tyrosinase with a molecular mass of 120 kDa exhibited an H_2_L_2_ tetramer structure. The H subunit is the tyrosinase domain that possesses multiple loops, α-helices, and β-strands, and the L subunit has a lectin-like fold containing β-strands. The active site of tyrosinase located in two antiparallel α-helices of the H subunit contains two copper ions, which engage in strong interaction with three Histidine residues^9,10^. Noteworthy, the structure and function of the active site are similarly preserved in various types of organisms^11^.

Accordingly, the identification and development of novel anti-tyrosinase agents with anti-melanogenesis potential as an added value are currently the subject of interest in medicine as well as the cosmetic and food industries. Several natural and synthetic compounds have been introduced as tyrosinase inhibitors including flavonoids, polyphenols, phenylpropanoids, thioamides, thioureas, and kojic acid derivatives^12,13^. However, these derivatives face a lot of challenges and certain safety risks, as a result developing novel and potent inhibitors with low toxicity is highly needed.

The quinoline framework is a well-known heterocyclic scaffold with a simple synthetic procedure that has drawn special interest because of its inherent and diverse biological response including antibacterial, antifungal, antimalarial, anthelmintic, anticonvulsant, cardiotonic, anti-inflammatory, and analgesic activities^14,15^. In addition, quinoline has been proven to be an excellent tyrosinase inhibitor. Jineol (**A**) dose-dependently inhibited mushroom tyrosinase activity with an IC_50_ of 39.46 ± 0.01 μM and 50.35 ± 0.05 μM in presence of L-tyrosine and L-Dopa as substrate, respectively. Jineol also reduced melanin content in melanoma cells by downregulating MITF expression through interference with ERK1/2 and p38 phosphorylation and suppressing the protein levels of tyrosinase, tyrosinase-related protein 1 (TYRP-1) and TYRP-2^16^. Also, 2-(4-Fluorophenyl)-quinazolin-4(3H)-one (compound **B**) was introduced as a highly potent tyrosinase inhibitor with quinoline structure which demonstrated good activity compared to arbutin as the positive control (IC_50_ = 180 μM)^17^. In another study, thio-quinazolinones conjugated to kojic acid (compound **C**) were developed, synthesized, and evaluated for their inhibitory activity against mushroom tyrosinase. The most potent compound showed significant tyrosinase inhibitory activity and demonstrated 68.99% melanin content at 8 µM. Docking study showed that the substitutions on aromatic ring provide optimum bulkiness to participate in the different forms of interaction including H-bound, in π-π stacking, and π-aryl interactions with the binding site of tyrosinase^18^.

**Figure 1:**
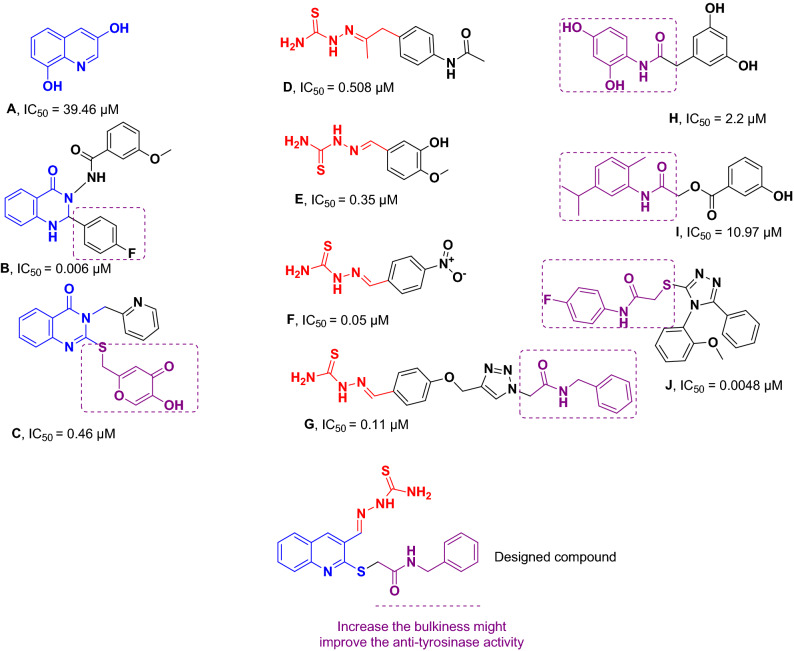
The rational design of the target compound based on previously reported tyrosinase inhibitors.

Thiosemicarbazide is known as a powerful anti-tyrosinase agent due to its capacity to reduce back o‐dopaquinone to L-Dopa and avoid melanin formation. Noteworthy it was well documented that thiosemicarbazide frequently appeared in tyrosinase inhibitors which are developed based on the structural modifications of linker type and length of L-tyrosine amino acid. In this context compounds, **D-G** bearing thiosemicarbazide moiety are good examples that showed high potency against tyrosinase (Fig. 1)^19–21^. Recently, compound **G** was developed as high potent tyrosinase inhibitor against tyrosinase with an IC_50_ value of 0.11 μM and 0.17 μM in the presence of L-tyrosine and L-Dopa as substrates, respectively. This side chain mimics the inherent structure of the native substrate and increases the affinity to tyrosinase.

Also, the biological activity of some designed compounds based on aryl-acetamide moieties is displayed in Fig. 1 (compound **H–J**)^22–24^. In silico studies confirmed that this linker is effectively involved in hydrogen bond interaction with the His residues and Cu ions of the tyrosinase active site and expanding the opportunity for further derivatization.

Regarding that thiosemicarbazide and aryl-acetamide presented in previous potent inhibitors and thioquinoline provide a suitable site to occupy the binding site and participate in several interactions with the enzyme, therefore, this study aimed to synthesize and evaluate the tyrosinase inhibitory potential of thiosemicarbazide-thioquinoline derivative bearing different aryl-acetamides. Moreover, the kinetics study and in silico evaluations were performed to determine the type of inhibition and binding pose within the enzyme. The cytotoxicity, as well as anti-melanogenesis potencies of the most potent derivative, were also executed.

## Results and discussion

### Chemistry

**Figure 2:**
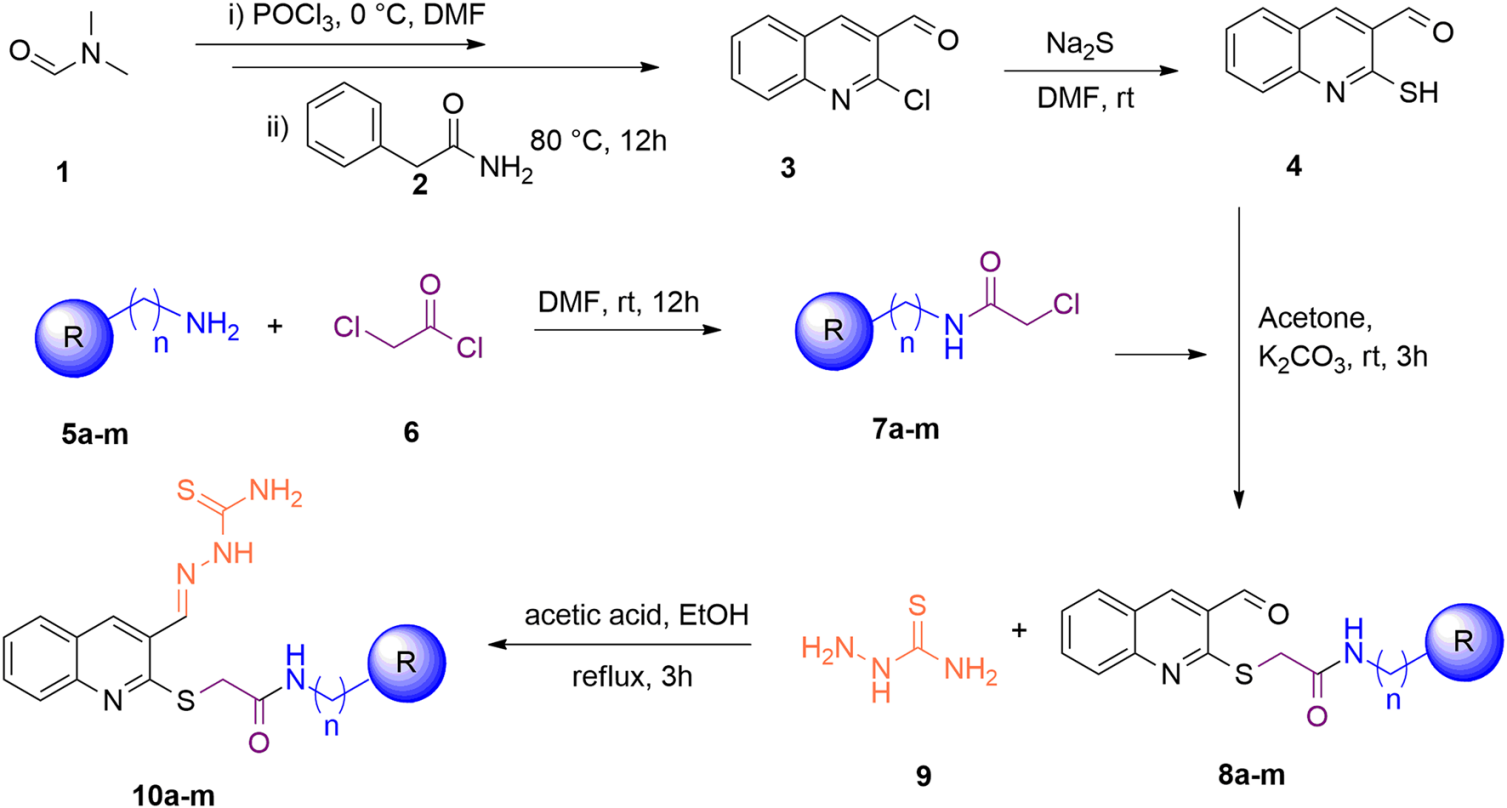
The synthetic path for the target compounds **10a-m**.

As presented in Fig. 2 to the mixture of N,N-dimethylformamide (**1**) and phosphoryl chloride in DMF at 0 °C, phenyl-acetamide (**2**) was added dropwise. After around 30 min, the mixture was heated to 80 °C for 12 h and the crude product was purified by recrystallization in ethanol to synthesize chloroquinoline-3-carbaldehyde (compound **3**)^25^.

Compound** 4** in turn was prepared from the reaction of sodium sulfide with compound **3** in DMF at room temperature for 2 h. Next, amine derivatives (**5a-m**) were cooled to 0 °C in DMF and chloroacethyl chloride (**6**) was added. The reaction mixture was then stirred at room temperature for 12 h then cold water was added. The resulting solid was washed with water three times with petroleum ether giving a solid and pure product (**7a-m**). The yield of each derivative were presented in Table 1.

**Table 1:** The synthetic results of **7a-m**.

Entry	Name of derivatives	Color	Yield (%)
7a	2-chloro-N-phenylacetamide	White solid	92
7b	2-chloro-N-(p-tolyl)acetamide	White solid	88
7c	2-chloro-N-(4-ethylphenyl)acetamide	White solid	83
7d	2-chloro-N-(4-methoxyphenyl)acetamide	White solid	78
7e	2-chloro-N-(4-methoxyphenyl)acetamide	Purple solid	76
7f	2-chloro-N-(4-nitrophenyl)acetamide	Yellow solid	89
7g	2-chloro-N-(4-chlorophenyl)acetamide	White solid	94
7h	2-chloro-N-(2,4-dichlorophenyl)acetamide	White solid	90
7i	2-chloro-N-(4-fluorophenyl)acetamide	White solid	89
7j	N-(2-bromophenyl)-2-chloroacetamide	White solid	86
7k	N-benzyl-2-chloroacetamide	Pale Yellow solid	80
7l	2-chloro-N-(4-methylbenzyl)acetamide	White solid	73
7m	2-chloro-N-(4-fluorobenzyl)acetamide	White solid	77

Next different bases, and solvents, as well as different temperatures, were examined to react compound **4** with **7a,** and finally, potassium carbonate as a base in acetone at 25 °C for 3 h was chosen to afford the **8a–m** (Table 2).

**Table 2:** Optimization of reaction conditions of **8a**.

Entry	Solvent	Base	Temperature (°C)	Yield (%)
1	DMF	Na_2_CO_3_	25	50
2	DMF	K_2_CO_3_	25	70
3	DMF	Na_2_CO_3_	70	55
4	DMF	K_2_CO_3_	70	73
5	EtOH	Na_2_CO_3_	25	–
6	EtOH	K_2_CO_3_	25	–
7	EtOH	K_2_CO_3_	Reflux	–
8	EtOH	NaOH	25	40
10	EtOH	NaOH	Reflux	50
11	Acetone	Na_2_CO_3_	25	75
12	Acetone	K_2_CO_3_	25	85
13	Acetone	Na_2_CO_3_	50	78
14	Acetone	K_2_CO_3_	50	70

In the final step, different solvents, catalysts, and temperatures were used to examine the synthetic yield of **8a**. As can be seen in Table 3, ethanol in presence of AcOH under the reflux conditions was chosen. As a result, **10a–m** were prepared through the reaction of thiosemicarbazide with the appropriate **8a–m** in presence of a catalytic amount of acetic acid in ethanol. The reaction mixture was refluxed for 3–4 h and then cooled to room temperature. The resulting precipitate was filtered, washed with ether, and recrystallized from ethanol to obtain the final product **10a–m** derivatives^26^.

**Table 3:** Optimization of reaction conditions of **10a**.

Entry	Solvent	Catalyst	Temperature (°C)	Yield (%)
1	EtOH	PTSA	25	60
2	EtOH	PTSA	50	70
3	EtOH	PTSA	Reflux	72
4	MeOH	PTSA	25	65
5	MeOH	PTSA	50	72
6	MeOH	PTSA	Reflux	76
7	EtOH	HCl	25	50
8	EtOH	HCl	50	60
10	EtOH	HCl	Reflux	65
11	MeOH	HCl	25	55
12	MeOH	HCl	50	67
13	EtOH	AcOH	25	70
14	EtOH	AcOH	50	75
15	EtOH	AcOH	Reflux	90
16	MeOH	AcOH	25	60
17	MeOH	AcOH	50	67
18	MeOH	AcOH	Reflux	76

The structures of all purified products, **10a–m,** were confirmed by IR, ^1^H NMR, ^13^C NMR, ESI–MS, and elemental analysis.

### Evaluation of tyrosinase inhibitory activity

The tyrosinase inhibitory activity of derivatives was determined by a colorimetric method and the results of the tyrosinase inhibitory assay were shown in Table 4 in the terms of IC_50_s.

**Table 4:** Tyrosinase inhibitory activities of **10a–m**.


Compound	R	IC_50_(µM) ± RSD^a^
**10a**		155.46 ± 4.39
**10b**		≥ 200 µM
**10c**		85.26 ± 2.94
**10d**		100.83 ± 1.27
**10e**		47.35 ± 0.13
**10f**		39.85 ± 0.07
**10g**		25.75 ± 0.19
**10h**		168.77 ± 3.60
**10i**		66.28 ± 0.15
**10j**		≥200
**10k**		61.96 ± 2.58
**10l**		78.54 ± 0.01
**10m**		53.34 ± 0.23
Kojic acid^b^		34.93 ± 0.06

50% inhibitory concentration (IC_50_).

^a^Values represent means ± RSD of 3 independent experiments.

^b^Kojic acid as the positive control.

As can be seen in Table 4, **10a** as an unsubstituted derivative (R = phenyl) exhibited weak tyrosinase inhibition with an IC_50_ value of 155.46 µM. Next, to improve the inhibitory activity, different substitutions on this ring were performed.

The incorporation *of para*-methyl as a small electron-donating group (**10b**, R = 4-methyl phenyl; IC_50_ ≥ 200 µM) deteriorated the activity while *para*-ethyl moiety as a bulk electron-donating group (**10c**, R = 4-ethyl phenyl; IC_50_ = 85.26 µM) improved the inhibition to around two-fold compared to **10a**. The same trend were seen in **10d** bearing spacious 2,3 dimethyl moiety with improved potency (**10d**, 2,3-methyl phenyl; IC_50_ = 100.83 µM). Substitution of the methoxy as an electron-donating group on the phenyl ring resulted in an around the three-fold amend of potency compared to **10a** (**10e**, R = 2,3-methyl phenyl; IC_50_ = 47.35 ± 0.13 µM). It seems that the presence of electron-donating heteroatom on the aromatic ring increased the inhibitory activities.

NO_2_ substitution (**10f**, R = 4-nitrophenyl) as strongly electron-withdrawing through both resonance and inductive effects demonstrated an IC_50_ value of 39.85 µM resulting in the second potent derivative.

The evaluations on **10g–j** as the halogen-substituted group exhibited that **10g** (R = 4-chlorophenyl) with an IC_50_ of 25.75 ± 0.19 μM was categorized as the top potent tyrosinase inhibitor in this group. Indeed addition of an extra Cl functional group at the *ortho* position of **10g** was inferior the activity (**10h**, 2,3-dichlorophenyl; IC_50_ = 168.77 µM). Although **10i** containing 4-fluorophenyl (IC_50_ = 66.28 µM) decreased the activity compared to its 4-chlorine counterpart, the activity was improved in comparison with **10a**. Assessments on bromine substituted moiety showed that the *ortho-*bromide group deteriorated the potency (**10j**, 2-bromophenyl; IC_50_ ≥ 200 µM).

Comparison of **10k** (R = benzyl; IC_50_ = 61.96 µM ) with **10a** (R = phenyl; IC_50_ = 155.46 µM) showed the important role of linker elongation so that **10k** disclosed around 2.5 fold improvement in the potency compared to **10a**. The same trend were seen in **10l** (R = 4-bromophenyl; IC_50_ = 78.54 µM) compared to (**10b**, 4-methylphenyl IC_50_ ≥ 200 µM) as well as **10m** (IC_50_ = 53.34 ± 0.23 µM) in comparesion with **10i** (IC_50_ = 66.28 ± 0.15 µM). From the screening data of the **10k–m** bearing benzyl group compared to their counterpart in the phenyl set (**10a–j**), superior inhibitory activity against tyrosinase was seen.

**Figure 3:**
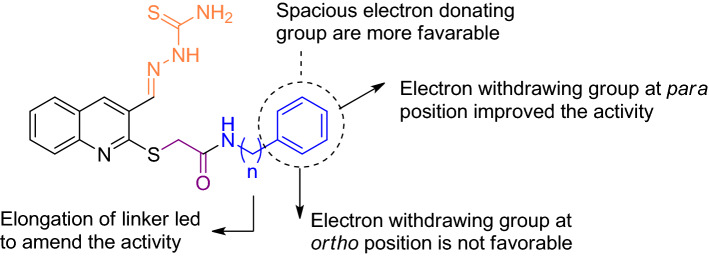
Summary of SAR of **10a-m** derivatives.

The summary of SAR of **10a–m** was presented in Fig. 3.

### Determining the inhibitory type of 10 g against mushroom tyrosinase

**Figure 4:**
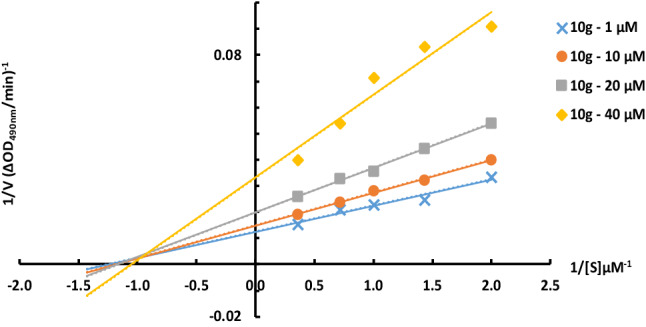
Lineweaver–Burk plot for mushroom tyrosinase enzyme inhibition by different concentrations of **10g** in the presence of L-Dopa. The reciprocal tyrosinase inhibitory activity was plotted against the reciprocal substrate concentration (double reciprocal plot, n = 3).

The enzyme inhibition mode by the most potent derivative, **10g**, was studied by Lineweaver–Burk plot analysis. The results are presented in Fig. 4. Lineweaver–Burk plots (plot of 1/V versus 1/[S]) for the inhibition of tyrosinase were obtained with several concentrations of **10g** (as the inhibitor) and L-Dopa (as the substrate). The plots of 1/V versus 1/[S] presented straight lines which crossed the x-axis at similar points. It was found that as the inhibitor concentration elevated, the value of *V*_*max*_ was reduced, but *K*_*m*_ was not affected by the concentration. Therefore, the results represented that compound **10g** is a noncompetitive inhibitor of mushroom tyrosinase. The obtained values for the *K*_*m*_ and *V*_*m*_ are summarized in Table 5.

**Table 5:** Kinetic parameters for the compounds **10g** against mushroom tyrosinase.

**10g**	*Vmax* (mM/min)	*Km* (mM)
1 µM	80.00	0.80
10 µM	67.57	0.85
20 µM	50.25	0.85
40 µM	3012	0.95

### Fluorescence quenching spectra of tyrosinase

**Figure 5:**
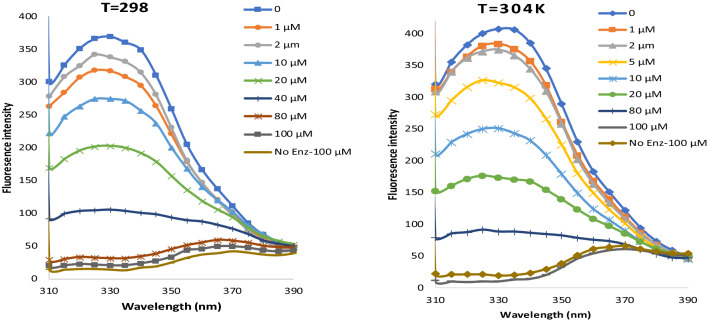
Fluorescence intensity of Enzyme in presence of different concentrations of inhibitor **10g**. (From top to down concentration of **10g** increased).

Tyrosinase intrinsic fluorescence was studied as a means of elucidating the inhibitory mechanism of **10g.** As shown in Fig. 5. tyrosinase emitted strong fluorescence with a maximum wavelength of 330 nm after being excited at 280 nm. Tyrosinase fluorescence intensity reduced as the concentration of inhibitor **10g** increased.

The quenching of the intrinsic fluorescence provided unambiguous evidence that the **10g** was capable of binding to tyrosinase, and the binding of **10g** to tyrosinase resulted in a change in the microenvironment around the fluorophore. Table 6 summarizes the K_sv_ values obtained from Eq. (1).

**Table 6:** The Stern–Volmer constants for the binding of **10g** with tyrosinase at 298 K and 304 K.

T (K)	Equation	K_sv_ (L.mol^–1^)	K_q_ (L⋅mol^–1^⋅s^–1^)	R^2^
298	y = 0.0669x + 0.8259	6.69 × 10^4^	6.69 × 10^12^	0.9946
304	y = 0.0514x + 1.0382	5.14 × 10^4^	5.14 × 10^12^	0.9719

**Figure 6:**
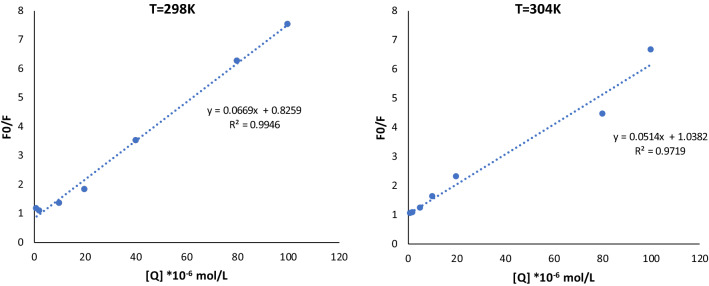
Sterm-Volmer plots for the quenching of tyrosinase by **10g** at 298 K and 304 K.

One reliable method for classifying the quenching process is to examine how temperature affects drug-enzyme interaction. The slope of the fluorescence curves in Fig. 6 was used to determine the fluorescence quenching constant, K_SV_, and after that k_q_ for temperatures of 298 K, and 304 K. From Table 6, it was observed that K_q_ of tyrosinase by inhibitor **10g** were all higher than 2 × 10^10^ L⋅mol^−1^⋅s^−1^. Since it has been reported, that kq in different types of quenchers with biopolymer is generally 2 × 10^10^ L⋅mol^-1^⋅s^-1^, compound **10g** is presumed to quench the intrinsic fluorescence of biomacromolecules through a static process. K_sv_ values for compound **10g** were inversely related to temperature, suggesting that **10g** and tyrosinase interact in a way that is impeded by static quenching.

### Binding constants and binding sites

If there were identical and independent binding sites in a protein during the process of static quenching, the binding constant (K_A_) and the binding site (n) might be calculated using the Double Logarithm of Log ((F_0_ – F)/F) = log K_A_ + n Log [Q] Eq. (2).

**Figure 7:**
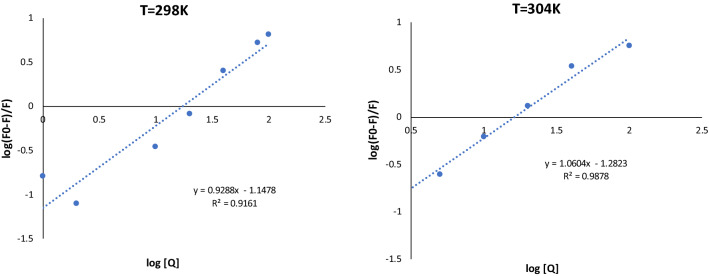
Double-Logarithmic plots of **10g** quenching effect on tyrosinase fluorescence at 298 K and 304 K.

As implied by Eq. (2), there is a straight-line relationship between log ((F_0_ − F)/F) versus log [Q], with the slope equal to n and the intercept equal to log K_A_ (Fig. 7). Table 7 displays that the n values for inhibitor-tyrosinase complexes were close to 1, indicating that a single inhibitor **10g** coupled to a single tyrosinase molecule. Additionally, when the temperature increased, the value of K_A_ decreased, which was consistent with the reliance of k_sv_ on temperature. The findings are consistent with a static quenching process.

**Table 7:** Binding parameters of the interaction of inhibitor **10g** with an enzyme derived from modified Stern–Volmer equation at 298 K and 304 K.

T/K	K_A_/(L mol^-1^)	n	R^2^
298	7.115 *10^4^	0.9288	0.9161
304	5.22*10^4^	1.0604	0.9878

### Molecular docking study

**Figure 8:**
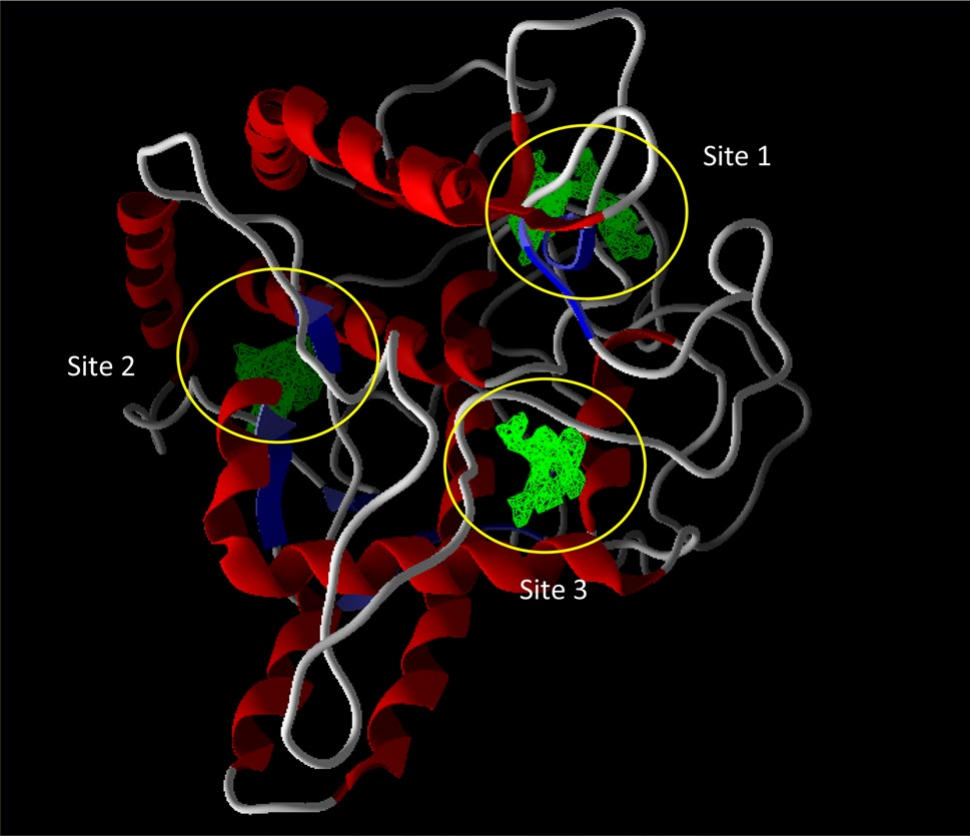
Potential binding sites for tyrosinase noncompetitive inhibitor colored as green.

**Figure 9:**
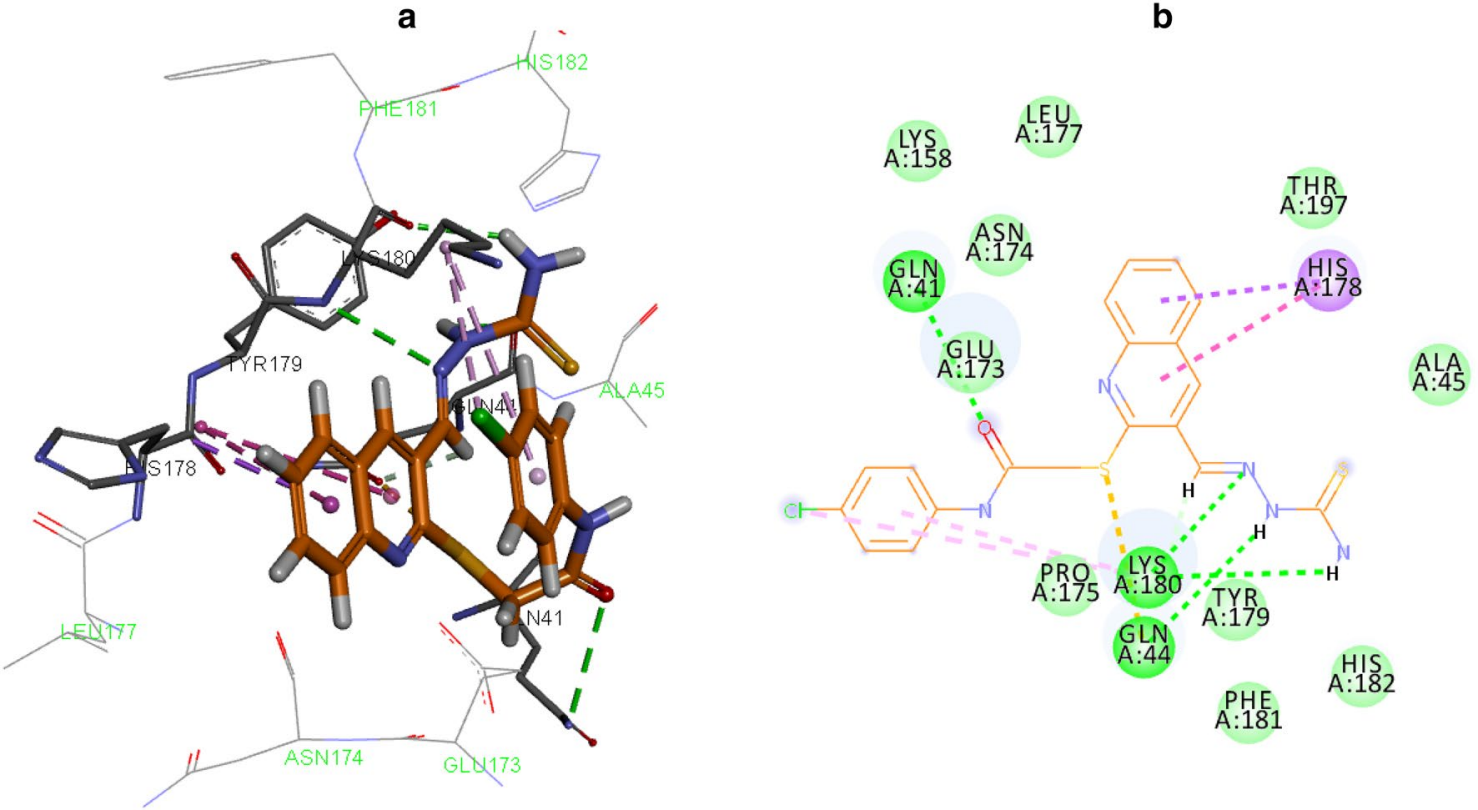
3D (a) and 2D (b) binding orientation and interactions of compound **10g** into the tyrosinase binding site.

The molecular binding analysis was then performed to gain insight into the interactions and binding mode of **10g** in the tyrosinase active site. First docking validation was performed through the docking of tropolone as a crystallographic inhibitor against tyrosinase (PDB code: 2Y9X). MoleDock scoring functions were examined and the RMSD value was less than 2.00 Å. According to the kinetic study, **10g** is a noncompetitive inhibitor and **10g** binds to the enzyme at a location other than the active site. As a result, MolDock cavity detection was applied to find the possible cavity of the enzyme. As presented in Fig. 8, three possible binding sites were detected on the surface of the enzyme which can be suitable for noncompetitive inhibition (regardless of the active site). Next **10g** as the potent inhibitor was docked on all of the potential binding sites of the enzyme. Considering the Moledock score and interactions, site 3 demonstrated the highest affinity in comparison to other identified sites (Fig. 9).

As can be seen in Fig. 8, compound **10g** fitted well in the tyrosinase allosteric site through various interactions including hydrogen bonding, electrostatic and hydrophobic interactions which enhanced its potency. The detailed interaction information of **10g** is presented in Table 8.

**Table 8:** Molecular docking results of compound **10g** with tyrosinase (PDB ID: 2Y9X).

Compound	MolDock score	Residues	Interaction type	Distance
		Gln41	H-bound	3.26
		Gln44	H-bound	2.97
		Gln44	Sulfur-X	2.96
		Lys180	H-bound	3.30
**10g**	− 137.007	Lys180	H-bound	2.30
		Lys180	Pi-alkyl	5.39
		Lys180	Alkyl	4.35
		His178	Pi-sigma	3.97
		His178	Amide pi-stacked	4.67
		His178	Amide pi-stacked	4.74

### Cell viability assessments

First, the toxicity of all derivatives against HEK 293 as a normal cell line was evaluated by MTT assay, and the results were presented in Table 9. The dose-dependent reduction of viability upon the increase in the concentration of derivatives can be seen.

**Table 9:** Cell viability of **10a–m** against HEK 293 cell line.

Cell line		Cell viability (%)^a^		
	**R**	16 µM	32 µM	64 µM
**10a**		90.19 ± 0.89	72.15 ± 2.13	65.82 ± 1.99
**10b**		96.22 ± 2.58	81.55 ± 2.91	79.43 ± 3.49
**10c**		82.55 ± 0.50	69.35 ± 1.81	58.33 ± 1.03
**10d**		87.52 ± 0.65	79.12 ± 0.62	71.39 ± 2.97
**10e**		85.59 ± 1.08	71.20 ± 3.86	61.99 ± 4.15
**10f**		97.67 ± 0.55	88.12 ± 4.05	80.05 ± 2.05
**10g**		92.71 ± 1.47	86.14 ± 2.17	72.54 ± 2.41
**10h**		96.91 ± 1.59	78.67 ± 2.19	67.88 ± 3.21
**10i**		75.30 ± 2.36	58.75 ± 5.50	46.59 ± 2.59
**10j**		91.15 ± 4.35	88.65 ± 2.31	79.90 ± 3.16
**10k**		77.28 ± 2.55	65.38 ± 3.49	51.11 ± 1.06
**10l**		75.12 ± 1.99	62.58 ± 1.58	50.01 ± 0.88
**10m**		95.47 ± 2.97	86.44 ± 1.17	71.25 ± 5.42

Doxorubicin as positive control exhibited an IC_50_ value of 1.3 ± 0.4 µM.

^a^Presented data are the mean (± S.E.M.) of three independent determinations.

MTT assessments of all derivatives at 16 µM exhibited no significant toxicity. The exception in this trend came back to **10i** (R = *para* fluorophenyl), **10k** (R = benzyl), and **10l** (R = *para*-methyl benzyl) with 75.30 ± 2.36%, 77.28 ± 2.55% and 75.12 ± 1.99% cell viability, respectively. Derivatives **10f**, **10g**, **10j** and **10m** exhibited limited toxicity (viability > 85% ) at 32 µM. Evaluation among the phenyl set of compounds exhibited that the incorporation of fluorine group at the *para* position of phenyl ring (**10b**) reduced the bioavailability to around 46.59% followed by compound **10c** bearing *para* ethyl moiety with 58.33 ± 1.03 cell viability at 64 µM. Among benzyl analogs, it was understood that elongation of the linker reduced the bioavailability. This trend can be seen in **10a** *vs* **10k** as well as **10b** *vs* **10l**. The exception in this trend came back to **10m** which showed 71.25% cell viability compared to **10i** 46.59%.

Next, the MTT assessment on **10g** as the most potent tyrosinase inhibitor on the A375 cell line was performed and this compound did not show significant toxicity up to 32 μM (Table. 10).

**Table 10:** Cell viability of **10g** on A375 cells.

	Cell viability (%)^[a]^		
Compound	16 µM	32 µM	64 µM
**10g**	93.69 ± 2.24	87.54 ± 1.53	61.17 ± 3.91

^[a]^ Presented data are the mean (± S.E.M.) of three independent determinations.

### Melanin content assay

**Figure 10:**
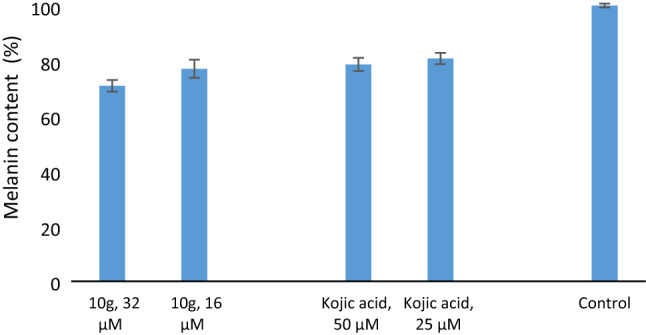
Effect of **10g** on melanin content in the A375 cell line.

The potency of **10g** to reduce the melanin content on the A375 cell line was evaluated. As can be seen in Fig. 10, **10g** reduced the melanin content in skin melanoma cells at tested concentrations compared to kojic acid as the positive control.

### ADME-Toxicity profiles and physicochemical properties

The physicochemical properties and pharmacokinetic profile of the new synthesis derivatives were summarized in Table 11^27^. The results good human intestinal absorption of all compounds which caused fast absorption from the intestine to the bloodstream. All derivatives showed low metabolism with cytochrome p400 with low clearance and limited rat acute toxicity (value less than 0.5 categorized as high toxic).

**Table 11:** ADMET prediction of **10a–m.**

Entry	Num. rotatable bonds	Num. H-bond acceptors	Num. H-bond donors	Molar refractivity	TPSA Å2	Log P o/w	GI-absorption	BBB permeability	Druglikness
**10a**	8	3	3	114.06	149.79	2.92	low	No	Yes (0 violation)
**10b**	8	3	3	119.02	149.72	3.29	low	No	Yes (0 violation)
**10c**	9	3	3	123.83	149.79	3.51	low	No	Yes (0 violation)
**10d**	8	3	3	123.99	149.79	3.63	low	No	Yes (0 violation)
**10e**	9	4	3	120.55	159.02	2.87	low	No	Yes (0 violation)
**10f**	9	5	4	121.30	199.45	0.56	low	No	Yes (0 violation)
**10g**	8	3	3	119.07	149.79	3.47	low	No	Yes (0 violation)
**10h**	8	3	3	124.08	149.79	4.04	low	No	Yes (0 violation)
**10i**	9	4	3	117.28	149.79	3.23	low	No	Yes (0 violation)
**10j**	8	3	3	121.76	149.79	3.58	low	No	Yes (0 violation)
**10k**	9	3	3	117.32	149.79	2.86	low	No	Yes (0 violation)
**10l**	9	3	3	122.29	149.79	3.20	low	No	Yes (0 violation)
**10m**	9	4	3	117.28	149.79	3.23	low	No	Yes (0 violation)

## Conclusion

Following our expertise in the rational design of tyrosinase inhibitors; herein, a series of thiosemicarbazide-thioquinoline derivatives bearing different aryl-acetamides were designed and synthesized. The most potent derivative **10g** bearing 4-chlorophenyl ring demonstrated an IC_50_ value of 25.75 ± 0.19 μM compared to that of kojic acid as the positive control (IC_50_ = 34.93 ± 0.06 μM). SAR study revealed that the presence of one electron-withdrawing group at the *para* position of the aromatic ring improved the activity compared to the rest of the derivatives. Moreover, it is worth mentioning that **10g** showed a noncompetitive inhibition mode of action in the enzymatic assay. In this context, in silico cavity detection was performed to extract the possible allosteric site and determine the binding pose of **10g** with the binding site. **10g** demonstrated several hydrophobic and hydrophilic interactions with the proposed binding site. In addition, cell toxicity assessments against HEK293 cell lines were executed and most derivatives exhibited no toxicity at 16 µM. Also, **10g** derivative was tested against A375 cell lines, and it exhibited a significant reduction of melanin content on A375 cell lines at tested concentrations. It can be understood that this set of compounds can serve as structural outlines to design and expand potential tyrosinase inhibitors.

## Material and methods

### Chemistry

All the reagents were purchased from commercial sources. ^1^H and ^13^C NMR spectra were determined by a Bruker Advance spectrometer 400 MHz spectrometer. All the chemical shifts were reported as (δ) values ppm. Multiplicities were indicated by s (singlet), d (doublet), t (triplet), q (quartet), m (multiplet), and coupling constant *J* was reported in hertz (Hz). CHNOS analysis was performed using Costech Company. IR spectra were obtained with a Nicolet, FR -IR Magna 550. Melting-point were also recorded using Kofler hot-stage apparatus. All the chemicals were purchased from Merck, Germany, and Sigma, Germany.

#### Synthesis of 2-chloroquinoline-3-carbaldehyde (3)

To stir DMF (3.6 mL, 46 mmol), 12.5 mL POCl_3_ (134 mmol) was added dropwise at 0 °C, then the mixture was allowed to stir for 30 min at room temperature. Next, acetanilide 1 (Compound **2**, 18.5 mmol) was added and the resulting mixture was heated for 12 h at 80–90 °C. The mixture was poured into ice-cold water and stirred for 10 min. The obtained yellow precipitate 2-chloroquinoline-3-carbaldehyde 2 after filtration, was washed with cold water, was dried and without purification^28^.

#### Synthesis of 2-mercaptoquinoline-3-carbaldehyde (4)

The reaction was initiated by stirring the mixture of 2-chloroquinoline-3-carbaldehyde 2 (Compound **3,** 1 mmol) and sodium sulfide (1 mmol) for 2 h at room temperature in dry DMF (5 mL). Then, the reaction mixture was poured into crushed ice and made acidic with acetic acid. The product was filtered off, washed with water, and dried to give the desired 2-mercaptoquinoline-3-carbaldehyde (Compound **4)** that was further purified by recrystallization in ethanol^28^.

#### Synthesis of 7a-m derivatives

A mixture of aniline derivatives (**5a–m**, 1 mmol) and chloro acetyl chloride 2 (compound **6,** 1 mmol) in DMF (5 ml) was stirred at room temperature for 30 min. Then, the obtained mixture was diluted with cold water, poured into ice, and formed a white precipitate that was filtered off. The residue was washed with cold water to obtain pure N‐phenyl‐2‐chloroacetamides^29^.

#### Synthesis of 8a-m derivatives

A mixture of 2-mercaptoquinoline-3-carbaldehyde (compound **4,** 1 mmol) and 2-chloro-N-substituted acetamide derivatives (**8a–m,** 1 mm mol) in dry acetone (10 mL) and anhydrous K_2_CO_3_ (1 mmol) was stirred at room temperature for 8 h, filtered and the solid product formed was crystallized from ethanol to give 2-((3-formylquinolin-2-yl)thio)-N-phenylacetamide derivatives^29^.

#### Synthesis of 10a-m derivatives

The appropriate thiosemicarbazide (compound **9**, 1 mmol) and the **8a–m** (1 mmol) were dissolved in ethanol (10 mL). To this solution, a catalytic amount of acetic acid was added. The reaction mixture was refluxed for 3–4 h and then cooled to room temperature. The resulting precipitate was filtered, washed with ether, and recrystallized from ethanol to obtain the corresponding final products (**10a–m**).

##### (E)-2-((3-((2-carbamothioylhydrazineylidene)methyl)quinolin-2-yl)thio)-N-phenylacetamide (10a)

Brown solid; Yield: 93%; MP = 180–182° C; IR (KBr, v_max_) 3400(NH_2_ ), 3150(NH), 3025 (C-H Aromatic), 2970(CH_2_ Aliphatic), 1675(C = O), 1520(C = N) Cm^-1^; ^1^H NMR (400 MHz,DMSO-*d*_*6*_) δ 11.86 (s, 1H, NH _Thio amid_), 10.44 (s, 1H, CH _Hydrazone_), 8.86 (s, 1H, NH _Amid_), 8.56, 7.95 (s, 2H, NH_2_), 8.45 (s, 1H, H_4_), 7.91(d, *J* = 7.90 Hz, 1H, H_5_), 7.83 (d, *J* = 8.40 Hz 1H, H_8_), 7.72 (t, *J* = 8.30 Hz, 1H, H_7_), 7.62 (d, *J* = 7.70 Hz, 1H, H_6_), 7.62 (d, *J* = 7.70 Hz, 2H, H_2_, , H_6_,), 7.53 (t, *J* = 7.50 Hz, 1H, H_6_), 7.31 (t, *J* = 7.90 Hz, 2H, H_3_, , H_5_,), 7.05 (t, *J* = 7.40 Hz, 1H, H_4_,), 4.25 (s, 2H, CH_2_) ppm. ^13^C NMR (101 MHz, DMSO-*d*_*6*_): δ 178.72, 167.22, 156.70, 147.28, 139.68, 137.88, 135.18, 131.38, 129.26, 128.86, 127.61, 126.69, 126.12, 125.80, 123.73, 119.52, 35.67 ppm; ESI–MS (C_19_H_17_N_5_OS_2_): calculated m/z 395.09 [M + H]^+^, observed m/z 395.12 [M + H]^+^Anal. Calcd: C_19_H_17_N_5_OS_2_ ; C, 57.70; H, 4.33; N, 17.71; Found C, 57.90; H, 4.50; N, 17.90.

##### (E)-2-((3-((2-carbamothioylhydrazono)methyl)quinolin-2-yl)thio)-N-(p-tolyl)acetamide (10b)

Cream solid;Yield:91%;MP = 180–185 °C; IR (KBr, v_max_) 3380(NH_2_), 3120(NH), 3040(C-H Aromatic), 2900(CH-Aliphatic), 1670(C = O), 1510 (C = N)Cm^-1^; ^1^H NMR (400 MHz,DMSO-d6) δ 11.81 (s, 1H, NH _Thio amid_), 10.34 (s, 1H, CH _Hydrazone_), 8.86 (s, 1H, NH _Amid_), 8.56, 7.91 (s, 2H, NH_2_), 8.45 (s, 1H, H_4_), 7.91(d, *J* = 7.80 Hz, 1H, H_5_), 7.83 (d, *J* = 8.40 Hz 1H, H_8_), 7.72 (t, *J* = 8.20 Hz, 1H, H_7_), 7.55–7.48 (m, 3H, H_6,_ H_2_, , H_6_,),7.10 (d, *J* = 8.30 Hz, 2H, H_3_, , H_5_, ), 4.25 (s, 2H, CH_2_), 2.23 (s, 3H, CH_3_) ppm. ^13^C NMR (101 MHz, DMSO-*d*_*6*_): δ 178.72, 166.96, 156.72, 147.28, 137.90, 137.17, 135.16, 132.63, 131.37, 129.62, 128.84, 127.62, 126.67, 126.12, 125.78, 125.70, 119.54, 35.64, 20.91 ppm; ESI–MS (C_20_H_19_N_5_OS_2_): calculated m/z 409.10 [M + H]^+^, observed m/z 440.8 [M + H]^+^, Anal. Calcd : C_20_H_19_N_5_OS_2_ ; C, 58.67; H, 4.68; N, 17.10; Found C, 58.69; H, 4.66; N, 17.12.

##### (E)-2-((3-((2-carbamothioylhydrazono)methyl)quinolin-2-yl)thio)-N-(4-ethylphenyl)acetamide (10c)

Brown solid;Yield:93%;MP = 178–180 °C IR (KBr, v_max_) 3390(NH_2_ ), 3130(NH), 3020(C-H Aromatic), 2975(CH_2_ Aliphatic), 1670(C = O), 1530 (C = N) Cm^-1^;^1^H NMR (400 MHz,DMSO-d6) δ 11.87 (s, 1H, NH _Thio amid_), 10.36 (s, 1H, CH _Hydrazone_), 8.85 (s, 1H, NH _Amid_), 8.56, 7.96 (s, 2H, NH_2_), 8.45 (s, 1H, H_4_), 7.91(d, *J* = 7.81 Hz, 1H, H_5_), 7.84 (d, *J* = 8.40 Hz 1H, H_8_), 7.72 (t, *J* = 8.30 Hz, 1H, H_7_), 7.57–7.50 (m, 3H, H_6,_ H_2_, , H_6_,),7.13 (d, *J* = 8.40 Hz, 2H, H_3_, , H_5_, ), 4.25 (s, 2H, CH_2_), 2.57–2.51(m.2H, CH_2ethyl_),1.14(t,* J* = 7.50 Hz,3H,CH_3_ethyl), ppm. ^13^C NMR (101 MHz, DMSO-*d*_*6*_): δ 178.73, 166.97, 156.72, 147.29, 139.12, 137.89, 137.36, 135.16, 131.37, 128.84, 128.43, 127.63, 126.67, 126.13, 125.79, 119.64, 35.62, 28.06, 16.17 ppm; Anal. Calcd : C_21_H_21_N_5_OS_2_ ; C, 59.55; H, 5.00; N, 16.53; Found C, 59.69; H, 5.16; N, 16.47.

##### (E)-2-((3-((2-carbamothioylhydrazono)methyl)quinolin-2-yl)thio)-N-(2,3-dimethylphenyl)acetamide (10d)

Brown solid;Yield:93%;MP = 185–187 °C IR (KBr, v_max_) 3430(NH_2_ ), 3140(NH), 3020(C-H Aromatic), 2975(CH_2_ Aliphatic) 1675(C = O), 1540 (C = N) Cm^-1^;^1^H NMR (400 MHz,DMSO-d6) δ 11.86 (s, 1H, NH _Thio amid_), 9.78 (s, 1H, CH _Hydrazone_), 8.89 (s, 1H, NH _Amid_), 8.55, 7.97 (s, 2H, NH_2_), 8.46 (s, 1H, H_4_), 7.95–7.89 (m, 2H, H_5,_ H_8_),7.77 (t, *J* = 8.2 Hz, 1H, H_7_), 7.56 (t, *J* = 7.70 Hz, 1H, H_6_), 7.11(d, *J* = 7.3 Hz, 1H, H_6_,), 7.07–6.96 (m, 2H, H_4_^,^_,_ H_5’_), 4.25 (s, 2H, CH_2_),2.21 (s.3H, CH_3_),2.02(s,3H,CH_3_) ppm. ^13^C NMR (101 MHz, DMSO-*d*_*6*_): δ 178.71, 167.17, 156.76, 147.38, 137.85, 137.42, 136.55, 135.07, 131.73, 131.36, 128.88, 127.72, 127.44, 126.70, 126.22, 125.86, 125.64, 123.86, 34.83, 20.59, 14.48 ppm; Anal. Calcd : C_21_H_21_N_5_OS_2_ ; C, 59.57; H, 5.03; N, 16.55; Found C, 59.57; H, 5.10; N, 16.47.

##### (E)-2-((3-((2-carbamothioylhydrazineylidene)methyl)quinolin-2-yl)thio)-N-(4-methoxyphenyl)acetamide (10e)

Cream solid;Yield:93%;MP = 191–193 °C; IR (KBr, v_max_) 3390(NH_2_ ), 3140 (NH), 3030 (C-H Aromatic), 2910 (CH-Aliphatic), 1680 (C = O), 1530(C = N)Cm^-1^;^1^H NMR (400 MHz,DMSO-d6) δ 11.85 (s, 1H, NH _Thio amid_), 10.30 (s, 1H, CH _Hydrazone_), 8.85 (s, 1H, NH _Amid_), 8.56, 7.74 (s, 2H, NH_2_), 8.45 (s, 1H, H_4_), 7.91(d, *J* = 7.70 Hz, 1H, H_5_), 7.85 (d, *J* = 8.30 Hz 1H, H_8_), 7.73 (t, *J* = 8.30 Hz, 1H, H_7_), 7.56–7.51 (m, 3H, H_6_ , H_2_, , H_6_,), 6.88 (d, *J* = 9.10 Hz, 2H, H_3_, , H_5_,), 4.23 (s, 2H, CH_2_), 3.71(s, 3H, CH_3_) ppm. ^13^C NMR (101 MHz, DMSO-d6): δ 178.72, 16.68, 156.74, 155.67, 147.30, 137.90, 135.13, 132.82, 131.37, 128.85, 127.64, 126.67, 126.13, 125.79, 121.10, 114.35, 55.59, 35.54 ppm; Anal. Calcd : C_20_H_19_N_5_O_2_S_2_; C, 56.45; H, 4.50; N, 16.46; Found C, 56.65; H, 4.70; N, 16.65.

##### (E)-2-((3-((2-carbamothioylhydrazineylidene)methyl)quinolin-2-yl)thio)-N-(4-nitrophenyl)acetamide (10f)

Pale yellow solid;Yield:93%;MP = 180–182 °C IR (KBr, v_max_) 3420 (NH_2_), 3150 (NH), 3020 (C-H Aromatic), 2965 (CH_2_ Aliphatic), 1670 (C = O), 1555–1350(NO_2_ ) Cm^-1^ ;^1^H NMR (400 MHz,DMSO-d_6_) δ, 11.12 (s, 1H, CH _Hydrazone_), 10.20 (s, 1H, NH _Amid_), 8.96 (s, 1H, H_4_), 8.24 (d, *J* = 9.30 Hz, 2H, H_3_, , H_5_,), 8.10 (d, *J* = 8.00 Hz, 1H, H_5_), 7.90 (d, *J* = 9.30 Hz, 2H, H_2_, , H_6_,), 7.87–7.82 (m, ,1H, H_8_), 7.79 (t, *J* = 8.10 Hz, 1H, H_7_), 7.59 (t, *J* = 8.10 Hz, 1H, H_6_), 4.25 (s, 2H, CH_2_) ppm. ^13^C NMR (101 MHz, DMSO-d6): δ 192.18, 168.76, 157.53, 148.76, 148.66, 146.52, 145.96, 142.56, 134.08, 130.11, 127.53, 127.19, 127.03, 125.58, 124.80, 119.09, 35.63 ppm; ESI–MS (C_19_H_16_N_6_O_3_S_2_): calculated m/z 440.07 [M + H]^+^, observed m/z 440.10 [M + H]^+^ ,Anal. Calcd : C_19_H_16_N_6_O_3_S_2_, C,51.81.29; H, 3.66; N, 19.08; Found C, 51.85; H, 3.64.; N, 19.15.

##### (E)-2-((3-((2-carbamothioylhydrazono)methyl)quinolin-2-yl)thio)-N-(4-chlorophenyl)acetamide (10g)

Brown solid;Yield:90%;MP = 185–191 °C; IR (KBr, v_max_) 3420(NH_2_ ), 3150(NH), 3030(C-H Aromatic), 2980 (CH_2_ Aliphatic), 1680(C = O), 1530 (C = N) Cm^-1^; ^1^H NMR (400 MHz,DMSO-d6) δ 11.87 (s, 1H, NH _Thio amid_), 10.60 (s, 1H, CH _Hydrazone_), 8.58 (s, 1H, NH _Amid_), 8.84, 8.45 (s, 2H, NH_2_), 7.95 (s, 1H, H_4_), 7.91(d, *J* = 7.77 Hz, 1H, H_5_), 7.80 (d, *J* = 8.50 Hz 1H, H_8_), 7.72 (t, *J* = 8.30 Hz, 1H, H_7_), 7.67 (d, 2H,* J* = 8.90 Hz,H_2_, , H_6_,), 7.52 (t, *J* = 8.00 Hz, 1H, H_6_)7.36 (d, *J* = 8.90 Hz, 2H, H_3_, , H_5_, ), 4.25 (s, 2H, CH_2_) ppm. ^13^C NMR (101 MHz, DMSO-*d*_*6*_): δ 178.72, 167.48, 156.60, 147.24, 138.65, 137.96, 135.30, 131.39, 129.18, 128.85, 127.56, 127.56, 127.26, 126.69, 126.09, 125.78, 121.04, 35.72 ppm; ESI–MS (C_19_H_16_ClN_5_OS_2_): calculated m/z 439.10 ,432.5[M + H]^+^and [M + H + 2]^+^,observed m/z 429.8,432.6 [M + H]^+^ and [M + H + 2]^+^, Anal. Calcd : C_19_H_16_ClN_5_OS_2_ ; C, 53.07; H, 3.75; N, 16.29; Found C, 53.19; H, 3.64; N,16.12C_19_H_16_N_5_OS_2_.

##### (E)-2-((3-((2-carbamothioylhydrazono)methyl)quinolin-2-yl)thio)-N-(2,4-dichlorophenyl)acetamide (10h)

Brown solid;Yield:86%;MP = 188–190 °C; IR (KBr, v_max_) 3440(NH_2_ ), 3160(NH), 3035 (C-H Aromatic), 2960 (CH_2_ Aliphatic), 1675 (C = O), 1540 (C = N) Cm^-1^;^1^H NMR (400 MHz,DMSO-d_6_) δ 11.88 (s, 1H, NH _Thio amid_), 9.96 (s, 1H, CH _Hydrazone_), 8.87 (s, 1H, NH _Amid_), 8.58,7.94 (s, 2H, NH_2_), 8.45 (s, 1H, H_4_), 7.88 (d, *J* = 8.40 Hz, 1H, H_5_), 7.81 (d, *J* = 8.80 Hz, 1H, H_6_,), 7.75 (d, *J* = 8.30 Hz, 1H, H_8_), 7.72 (t, *J* = 8.10 Hz, 1H, H_7_), 7.63 (d,^4^* J* = 2.4 Hz, 1H, H_3_,), 7.54 (t, *J* = 7.80 Hz, 1H, H_6_), 7.38 (dd, ^3^*J*_C-H_ = 8.80 Hz,^4^*J*_C-H_ = 2.50 Hz, 1H, H_5_,), 4.33 (s, 2H, CH_2_) ppm. ^13^C NMR (101 MHz, DMSO-d_6_): δ 178.71, 168.03, 156.30, 147.27, 137.90, 135.49, 134.58, 131.39, 129.45, 129.39, 129.33, 128.84, 128.08, 127.71, 126.79, 126.73, 126.48, 126.16, 125.87, 34.91 ppm;Anal. Calcd for C_19_H_15_C_l2_N_5_OS_2_ ; C, 49.14; H, 3.26; N, 15.08; Found ; C, 49.34; H, 3.45; N, 15.30.

##### (E)-2-((3-((2-carbamothioylhydrazono)methyl)quinolin-2-yl)thio)-N-(4-fluorobenzyl)acetamide (10i)

Brown solid;Yield:89%;MP = 186–188 °C; IR (KBr, v_max_) 3410(NH_2_ ), 3130(NH), 3060(C-H Aromatic), 2975 (CH_2_ Aliphatic), 1670(C = O), 1520 (C = N) Cm^-1^;^1^H NMR (400 MHz,DMSO-d_6_) δ 11.86 (s, 1H, NH _Thio amid_), 8.86 (s, 1H, CH _Hydrazone_), 8.76 (t, *J* = 6.10 Hz, 1H, NH _Amid_), 8.44 (s, 1H, H_4_), 8.56, 7.99 (s, 2H, NH_2_), 7.91(d, *J* = 8.00 Hz, 1H, H_5_), 7.78–7.68 (m, 2H, H_6_ , H_8_), 7.55 (t, *J* = 8,10 Hz, 1H, H_7_), 7.25–7.20 (m, 2H, H_2_, , H_6_,), 6.96 (t, *J* = 8,90 Hz, 2H, H_3_, , H_5_,), 4.11 (s, 2H, CH_2_) ppm. ^13^C NMR (101 MHz, DMSO-d_6_): δ 178.70, 168.21, 161.48(d, ^1^*J*_*CF*=_241), 156.59, 147.31, 137.85, 135.98, 135.94, 134.93, 131.19, 129.49, 129.41, 128.78, 127.80, 126.64, 126.22, 125.81, 115.33, 115.12, 42.24, 34.25 ppm;Anal. Calcd for C_20_H_18_FN_5_OS_2_: C, 56.19; H, 4.24; N, 16.38;Found; C, C, 56.40; H, 4.44; N, 16.59.

##### (E)-N-(2-bromophenyl)-2-((3-((2-carbamothioylhydrazono)methyl)quinolin-2-yl)thio)acetamide (10j)

Brown solid; Yield: 93%; MP = 181–183° C; IR (KBr, v_max_) 3380(NH_2_), 3140(NH), 3030 (C-H Aromatic), 2965(CH_2_ Aliphatic), 1655(C = O), 1540(C = N) Cm^-1^; ^1^H NMR (400 MHz,DMSO-*d*_*6*_) δ 11.85 (s, 1H, NH _Thio amid_), 10.52 (s, 1H, CH _Hydrazone_), 8.86 (t, *J* = 6.10 Hz, 1H, NH _Amid_), ), 8.57, 7.95 (s, 2H, NH_2_), 8.45 (s, 1H, H_4_), 7.91(d, *J* = 7.70 Hz, 1H, H_5_), 7.82 (d, *J* = 8,40 Hz, 1H, H_8_), 7.72 (t, *J* = 8,30 Hz, 1H, H_7_), 7.67–7.61 (m, 2H, H_2_, , H_6_,), 7.53 (t, *J* = 7.50 Hz, 1H, H_6_), 7.15 (t, *J* = 8,90 Hz, 2H, H_3_, , H_5_,), 4.20 (s, 2H, CH_2_), ppm. ^13^C NMR (101 MHz, DMSO-*d*_*6*_): δ 178.71, 167.64, 156.25, 147.32, 137.87, 136.62, 135.39, 133.10, 131.40, 128.84, 128.54, 127.83, 127.16, 126.80, 126.32, 126.20, 125.89, 117.12, 34.86 ppm; Anal. Calcd : C_19_H_16_BrN_5_OS_2_ ; C, 48.10; H, 3.40; N, 14.76; Found C, 48.30; H, 3.60; N, 14.95.

##### (E)-N-benzyl-2-((3-((2-carbamothioylhydrazono)methyl)quinolin-2-yl)thio)acetamide (10k)

Brown solid;Yield:94%;MP = 183–185 °C; IR (KBr, v_max_) 3410(NH_2_ ), 3160(NH) , 3045(C-H Aromatic), 2975 (CH_2_ Aliphatic) 1655 (C = O), 1520 (C = N) Cm^-1^;^1^H NMR (400 MHz,DMSO-d6) δ 11.84 (s, 1H, NH _Thio amid_), 8.88 (s, 1H, CH _Hydrazone_), 8.75 (t, *J* = 5.80 Hz, 1H, NH _Amid_), 8.54, 7.98 (s, 2H, NH_2_), 8.45(s, 1H, H_4_), 7.92(d, *J* = 8.00 Hz, 1H, H_5_), 7.82 (d, *J* = 8.30 Hz 1H, H_8_), 7.74 (t, *J* = 8,00 Hz, 1H, H_7_), 7.57 (d, *J* = 7.20 Hz, 1H, H_6_), 7.23–7.13 (m, 5H, H _phenyl_), 4.11 (s, 2H, CH_2_), ppm. ^13^C NMR (101 MHz, DMSO-*d*_*6*_): δ 178.72, 168.15, 156.64, 147.36, 139.75, 137.81, 134.87, 131.24, 128.80, 128.57, 127.86, 127.45, 127.08, 126.66, 126.25, 125.84, 42.92, 34.22 ppm; Anal. Calcd : C_20_H_19_N_5_OS_2_ ; C, 58.66; H, 4.68; N, 17.10; Found C, 58.54; H, 4.57; N, 17.24.

##### (E)-2-((3-((2-carbamothioylhydrazineylidene)methyl)quinolin-2-yl)thio)-N-(4-methylbenzyl)acetamide (10l)

Brown solid; Yield: 93%; MP = 180–182° C; IR (KBr, v_max_) 3370 (NH_2_ ), 3120 (NH), 3025 (C-H Aromatic), 2970(CH_2_ Aliphatic), 1675(C = O),1510 (C = N) Cm^-1^; ^1^H NMR (400 MHz,DMSO-*d*_*6*_) δ 11.85 (s, 1H, NH _Thio amid_), 8.87 (s, 1H, CH _Hydrazone_), 8.69 (t, *J* = 5.90 Hz, 1H, NH _Amid_), 8.54, 7.98 (s, 2H, NH_2_), 8.44 (s, 1H, H_4_), 7.92(d, *J* = 8.00 Hz, 1H, H_5_), 7.78 (d, *J* = 8.20 Hz 1H, H_8_), 7.77–7.69 (m, 1H, H_7_), 7.56 (t, *J* = 7.90 Hz, 1H, H_6_), 7.09 (d, *J* = 7.80 Hz, 2H, H_2_, , H_6_,), 6.98 (d, *J* = 7.80 Hz, 2H, H_3_, , H_5_,), 4.26 (d, *J* = 5.90 Hz, 2H, CH_2_-NH), 4.11 (s, 2H, CH_2_), 2.22(s, 3H, CH_3_) ppm. ^13^C NMR (101 MHz, DMSO-*d*_*6*_): δ 178.70, 171.11, 168.05, 156.64, 147.35, 137.79, 136.69, 136.10, 134.85, 131.19, 129.13, 128.78, 127.86, 127.50, 126.64, 126.22, 125.82, 42.69, 34.24, 21.13 ppm; Anal. Calcd : C_21_H_21_N_5_OS_2_ ; C, 59.55; H, 5.00; N, 16.54; Found C, 59.75; H, 5.20; N, 16.74.

##### (E)-2-((3-((2-carbamothioylhydrazono)methyl)quinolin-2-yl)thio)-N-(4-fluorobenzyl)acetamide (10m)

Brown solid;Yield:89%;MP = 186–188 °C; IR (KBr, v_max_) 3410(NH_2_ ), 3130(NH), 3060(C-H Aromatic), 2975 (CH_2_ Aliphatic), 1670(C = O), 1520 (C = N) Cm^-1^;^1^H NMR (400 MHz,DMSO-d_6_) δ 11.86 (s, 1H, NH _Thio amid_), 8.86 (s, 1H, CH _Hydrazone_), 8.76 (t, *J* = 6.10 Hz, 1H, NH _Amid_), 8.44 (s, 1H, H_4_), 8.56, 7.99 (s, 2H, NH_2_), 7.91(d, *J* = 8.00 Hz, 1H, H_5_), 7.78–7.68 (m, 2H, H_6_ , H_8_), 7.55 (t, *J* = 8,10 Hz, 1H, H_7_), 7.25–7.20 (m, 2H, H_2_, , H_6_,), 6.96 (t, *J* = 8,90 Hz, 2H, H_3_, , H_5_,), 4.11 (s, 2H, CH_2_) ppm. ^13^C NMR (101 MHz, DMSO-d_6_): δ 178.70, 168.21, 161.48(d, ^1^*J*_*CF*=_241), 156.59, 147.31, 137.85, 135.98, 135.94, 134.93, 131.19, 129.49, 129.41, 128.78, 127.80, 126.64, 126.22, 125.81, 115.33, 115.12, 42.24, 34.25 ppm;Anal. Calcd for C_20_H_18_FN_5_OS_2_: C, 56.19; H, 4.24; N, 16.38;Found; C, C, 56.40; H, 4.44; N, 16.59.

### Tyrosinase assay

Mushroom tyrosinase (EC 1.14.18.1) (Sigma Chemical Co.) was assayed using L-Dopa as the substrate as reported in our previous studies with some modifications^30–32^. The enzyme diphenolase activity was monitored spectrophotometrically by observing dopachrome formation at 490 nm. All the test samples were first dissolved in DMSO at 10 mM and diluted to the required concentrations. Initially, in a 96-well microplate, 10 µl of test samples were added to 160 µl of 50 mM phosphate buffer (pH = 6.8) and then 10 µl tyrosinase (500 U mL^−1^) was added. After the mixture was pre-incubated at 28 °C for 20 min, 20 µl of L-Dopa solution (7 mM) was added to the mixture. After 10 min incubation absorbance of samples was measured. DMSO without test compounds was used as the control, and kojic acid was used as a positive control. Each assay was conducted as three separate replicates. The inhibitory activity of the tested compounds was expressed as the concentration that inhibited 50% of the enzyme activity (IC_50_). The percentage inhibition ratio was calculated according to the following equation:

$${\text{Inhibition }}\left( \% \right) = {1}00*\left( {{\text{Abs}}_{{{\text{control}}}} - {\text{Abs}}_{{{\text{compound}}}} } \right)/{\text{Abs}}_{{{\text{control}}}}$$

### Enzyme kinetic studies

The kinetic study for the inhibition of tyrosinase by compound **10g** was carried out using four different concentrations of inhibitor (1, 10, 20, and 40 µM) against tyrosinase with different concentrations of L-Dopa (0.25, 0.5, 0.75, and 1 mM) as the substrate. The Lineweaver–Burk reciprocal plot was provided by plotting 1/V against 1/[S] at variable concentrations of the substrate L-Dopa (0.25, 0.5, 0.75, and 1 mM) The inhibition constant *K*_*i*_ was achieved by the plot of slopes versus the corresponding concentrations of the compound **10g**^24^.

### Fluorescence spectroscopy

The fluorescent measurements were carried out in each well of the black 96 well plate by adding various concentrations of inhibitor **10g**, (Q = 5, 10, 20, 40, and 100 µM) to a constant concentration of tyrosinase (273 IU/mL). Next, phosphate buffer solution (0.05 M, pH = 6.8) was used to bring the capacity of each well to 200 µL. Before measurements, all solutions were properly mixed and allowed to stand for 20 min at 298 K and 304 K. A fluorescence spectrometer (BioTek’s multi-mode plate reader, USA) was used to measure the fluorescence spectra. The bandwidths of excitation and emission were both set at 5 nm, and fluorescence emission spectra were captured spanning the wavelength range of 310–400 nm at an excitation wavelength of 280 nm.

The linear Stern–Volmer Eq. (1) was used to examine the fluorescence quenching:

1 $$\frac{F0}{F}=1+kq \tau 0 \left[Q\right]=1+kSV [Q]$$

where, F and F_0_ represent the fluorescence intensity in the presence or absence of inhibitors, respectively. The bimolecular quenching constant is denoted by the symbol k_q_, whereas the average lifespan of a biomolecule without a quencher is denoted by the symbol τ_0_, and its value is 10^−8^ s. [Q] refers to the inhibitor concentration (µM). K_SV_ which is calculated by k_q_ . τ_0._ is the fluorescence quenching constant.

In order to calculate the apparent binding constant and binding fraction of fluorophore group of tyrosinase accessible to inhibitor **10g**, Stern–Volmer Eq. (1) should be modified to Eq. (2):

2 $${\text{Log }}\left( {\left( {{\text{F}}_{0} {-}{\text{ F}}} \right)/{\text{F}}} \right) \, = {\text{ log K}}_{{\text{A}}} + {\text{ n Log }}\left[ {\text{Q}} \right]$$

In this equation, K_A_ indicates either the modified apparent binding constant or the Stern–Volmer quenching constant. n represents the fraction of fluorophore accessible to the quencher. The intercept of the curve that is obtained by plotting log (F_0_-F/F) versus log [Q] is log K_A_, and the slope of the curve is n. Therefore, the intercept and slope data may be used to calculate the values of K_A_ and n.

### In silico studies

The molecular docking studies of the most potent inhibitor were performed against tyrosinase (PDB code: 2Y9X) to observe the binding orientation and interactions using the MolDock program. The 3D crystal structure of tyrosinase was retrieved from Protein Data Bank. Water molecules and the cognate ligand (tropolone) were removed from the receptor and the hydrogen atoms were added and non-polar hydrogens were merged into related atoms of the receptor via protein preparation of MolDock software. Moldock scoring function and MolDock SE algorithm of the program were used for re-dock tropolone inside the enzyme with a binding site radius of 9 Å. All other options were set as default. **10g** were drawn using Hyperchem and subjected to energy minimization using MM^+^ and AM1 algorithms. Similarly, the MolDock program was applied for doing docking analyses of **10g**. The top-score binding pose was analyzed with Discovery Studio Visualizer^18,21,31^.

### MTT assay for cell viability

The cytotoxic activity of all derivatives against the HEK 293 cell line and **10g** against A375 (at a density of 5 × 10^3^ cells/ml) using 3-(4,5-dimethylthiazol-2-yl)-2,5- diphenyltetrazolium bromide (MTT) assay were performed. Cells were grown at 37 ºC in the presence of CO_2_ 5% in DMEM (Gibco BRL, Grand Island, NY, USA), 10% fetal bovine serum (FBS, Gibco BRL), and penicillin/streptomycin (100 IU/mL and 100 µg/mL, respectively). Next cells were seeded in a 96-well plate and incubated at 37 $^\circ{\rm C}$ with derivatives at different concentrations for 48 h. Following the treatment, cells were incubated with MTT (0.5 mg/ml) at 37 $\mathrm{^\circ{\rm C} }$ for 3 h. The MTT-containing medium was then removed, and 100 μl of DMSO was added to each well, mixed thoroughly with a 10 min shake to dissolve formazan crystals. The absorbance of each well was measured at 540 nm^33^.

### Determination of melanin content

The assay was performed according to previously reported procedures. In detail, A375 cells were seeded in six-well plates (2.0 × 10^5^ cells/well). After 24 h, the medium was replaced by a fresh one and treated with **10g** at different concentrations and the plate was incubated for an extra 48 h. After that cells were washed twice with PBS and harvested using 0.25 M trypsin, then dissolved in 300 μl of 1 N NaOH/10% DMSO buffer and boiled for 2 h at 80 ºC to solubilize the melanin. The absorbance of the supernatant was measured at 470 nm in a microplate reader. The obtained results were normalized using total protein content. Kojic acid was used as a positive^30,34^.

## Supplementary Information

Supplementary Information.

## Data Availability

The datasets generated and/or analysed during the current study are available in the Worldwide Protein Data Bank (wwPDB) repository. (http://www.rcsb.org).
