# Supplementary material for: Thioquinoline derivatives conjugated to thiosemicarbazide as potent tyrosinase inhibitors with anti-melanogenesis properties
Source: Sci Rep. 2023 Feb 13;13:2578. doi: 10.1038/s41598-023-28852-1 (PMC9925432; doi:10.1038/s41598-023-28852-1)
Supplement: Supplementary file 1 — Supplementary Information. [file 41598_2023_28852_MOESM1_ESM.docx]

*Fig S1. (E)-2-((3-((2-carbamothioylhydrazineylidene)methyl)quinolin-2-yl)thio)-N-phenylacetamide (10a)*

*
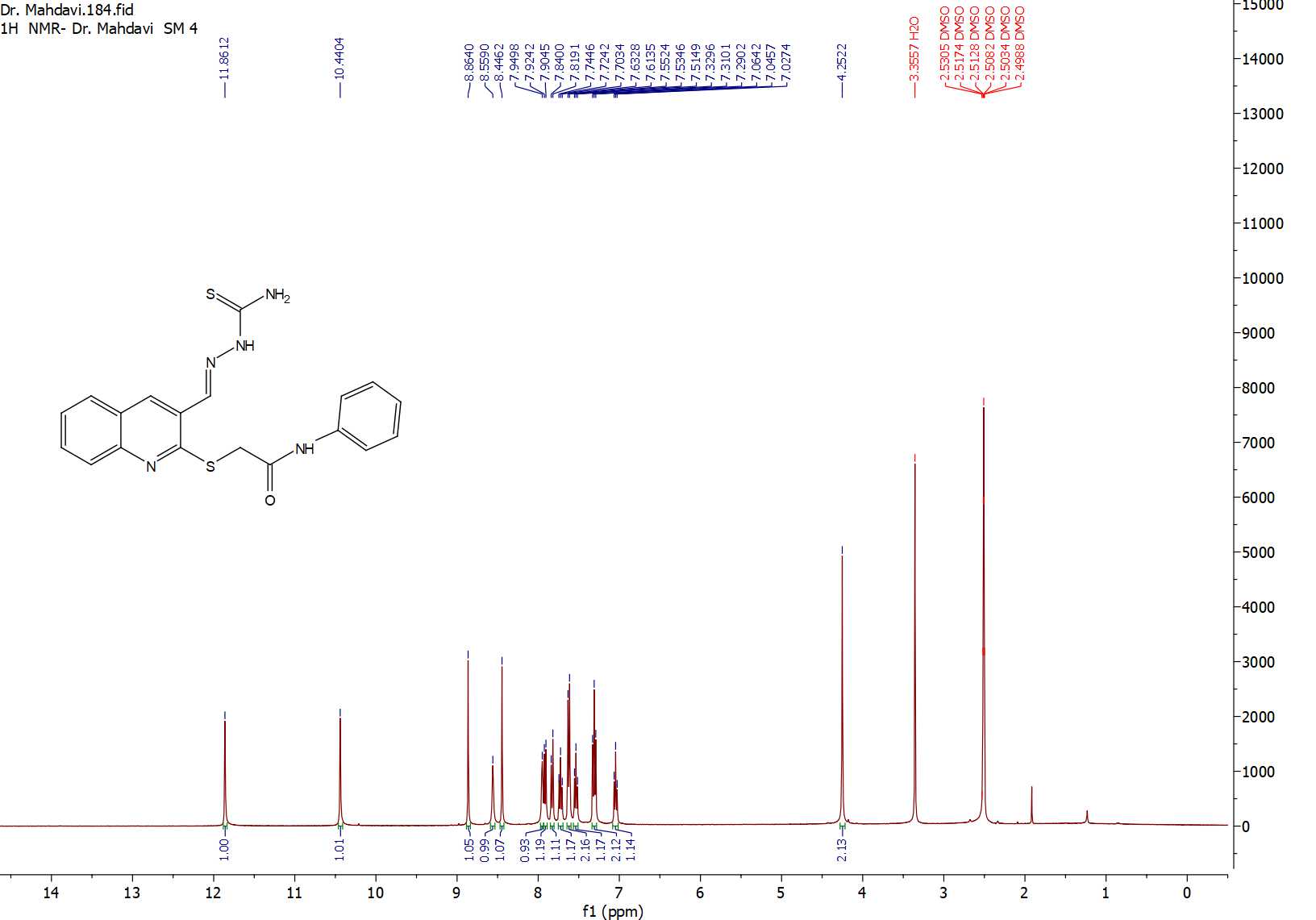
*

*
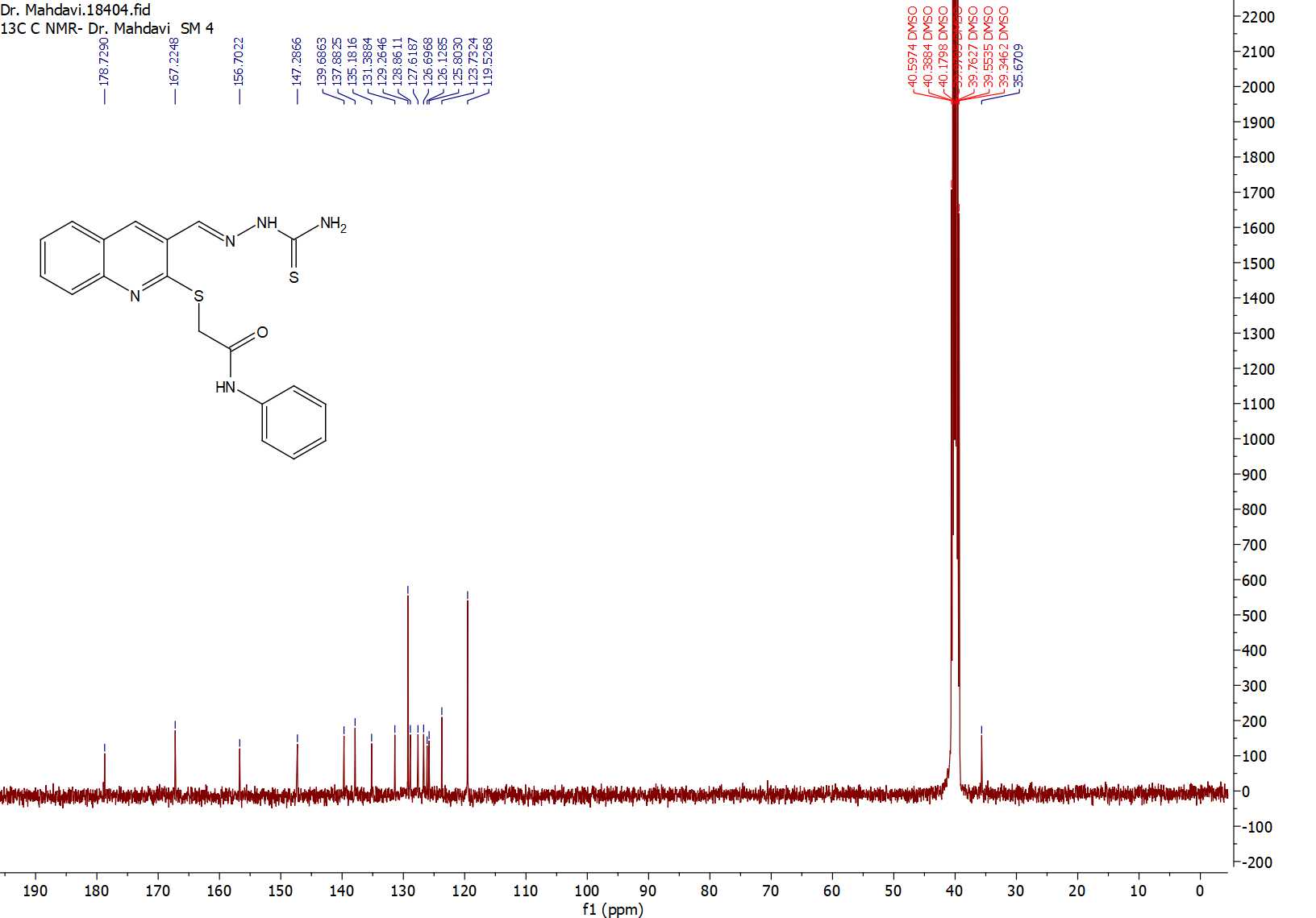
*

*Fig S2. (E)-2-((3-((2-carbamothioylhydrazono)methyl)quinolin-2-yl)thio)-N-(p-tolyl)acetamide (10b)*

*
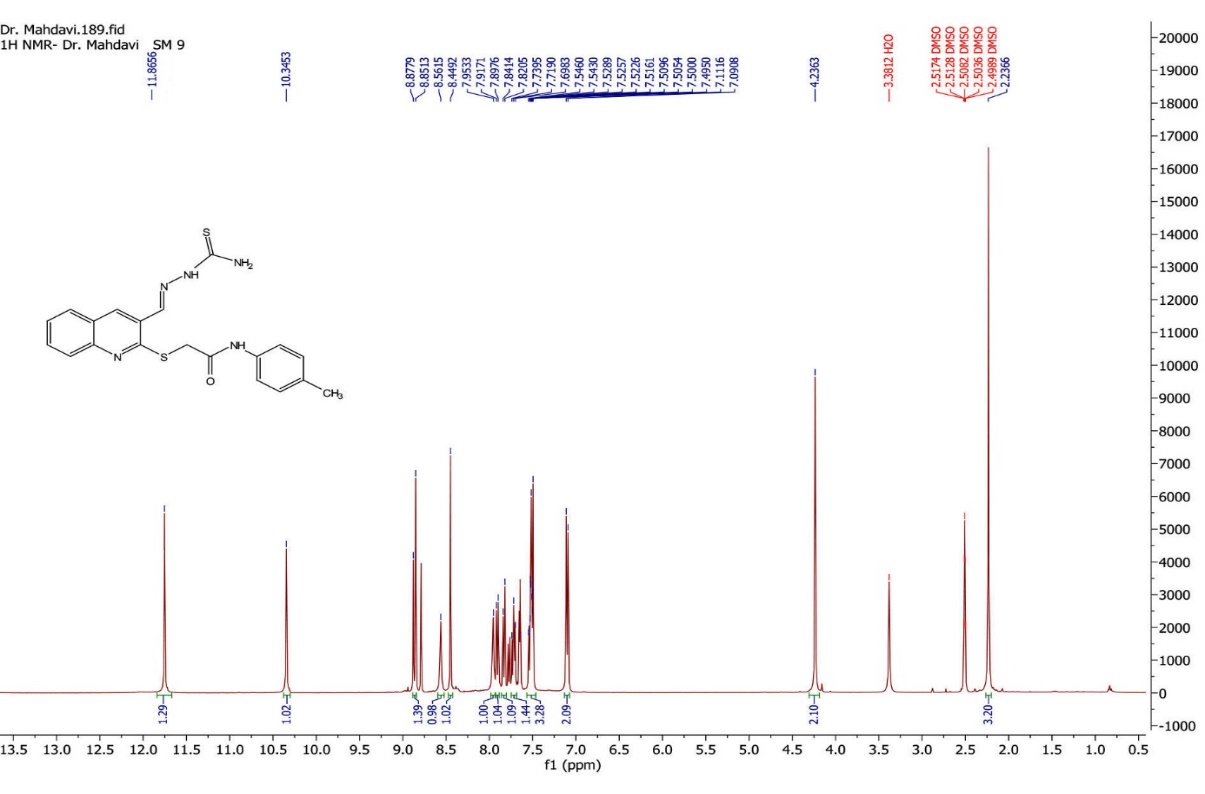
*

*
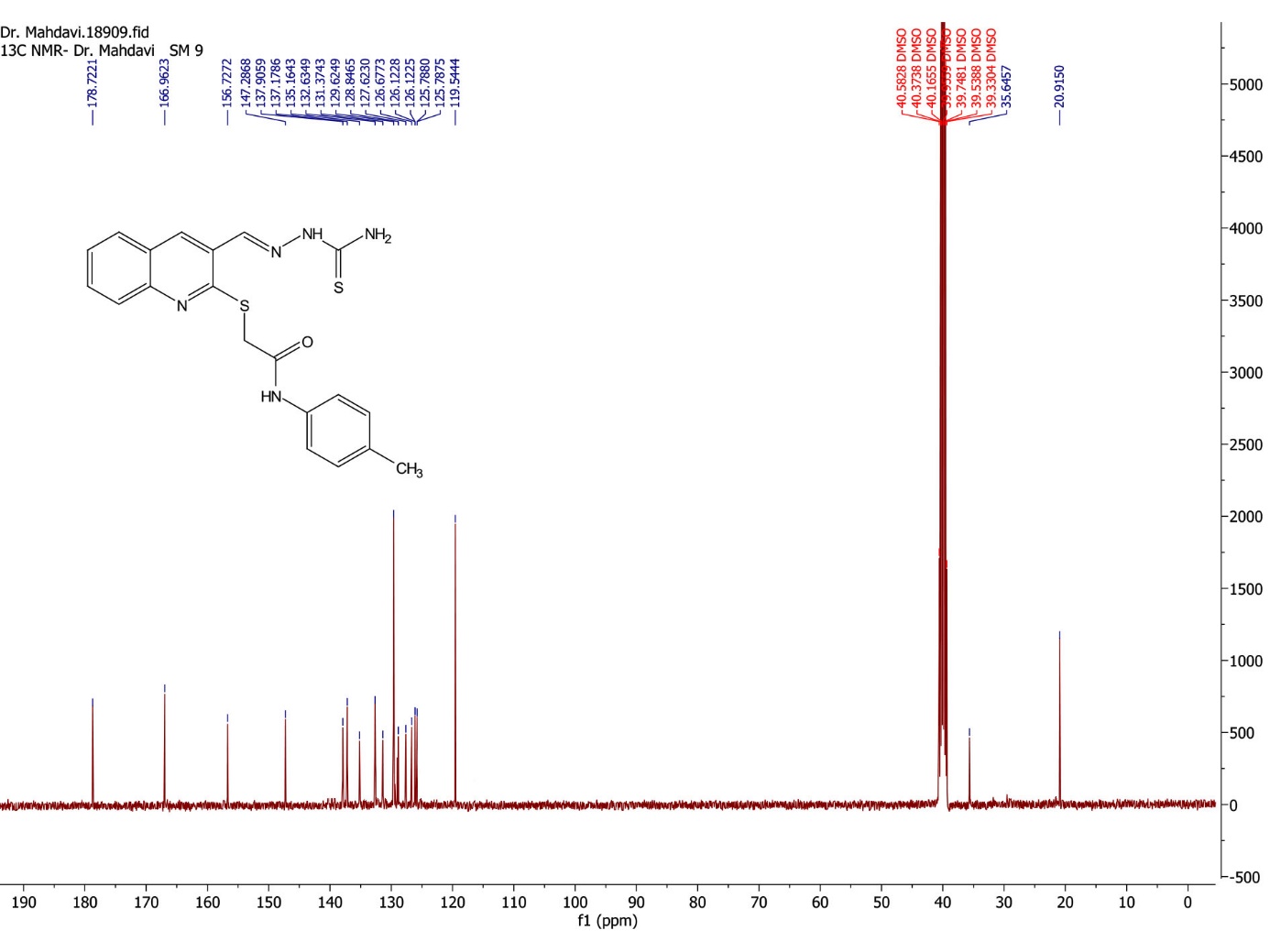
*

*Fig S3. (E)-2-((3-((2-carbamothioylhydrazono)methyl)quinolin-2-yl)thio)-N-(4-ethylphenyl)acetamide (10c)*

*
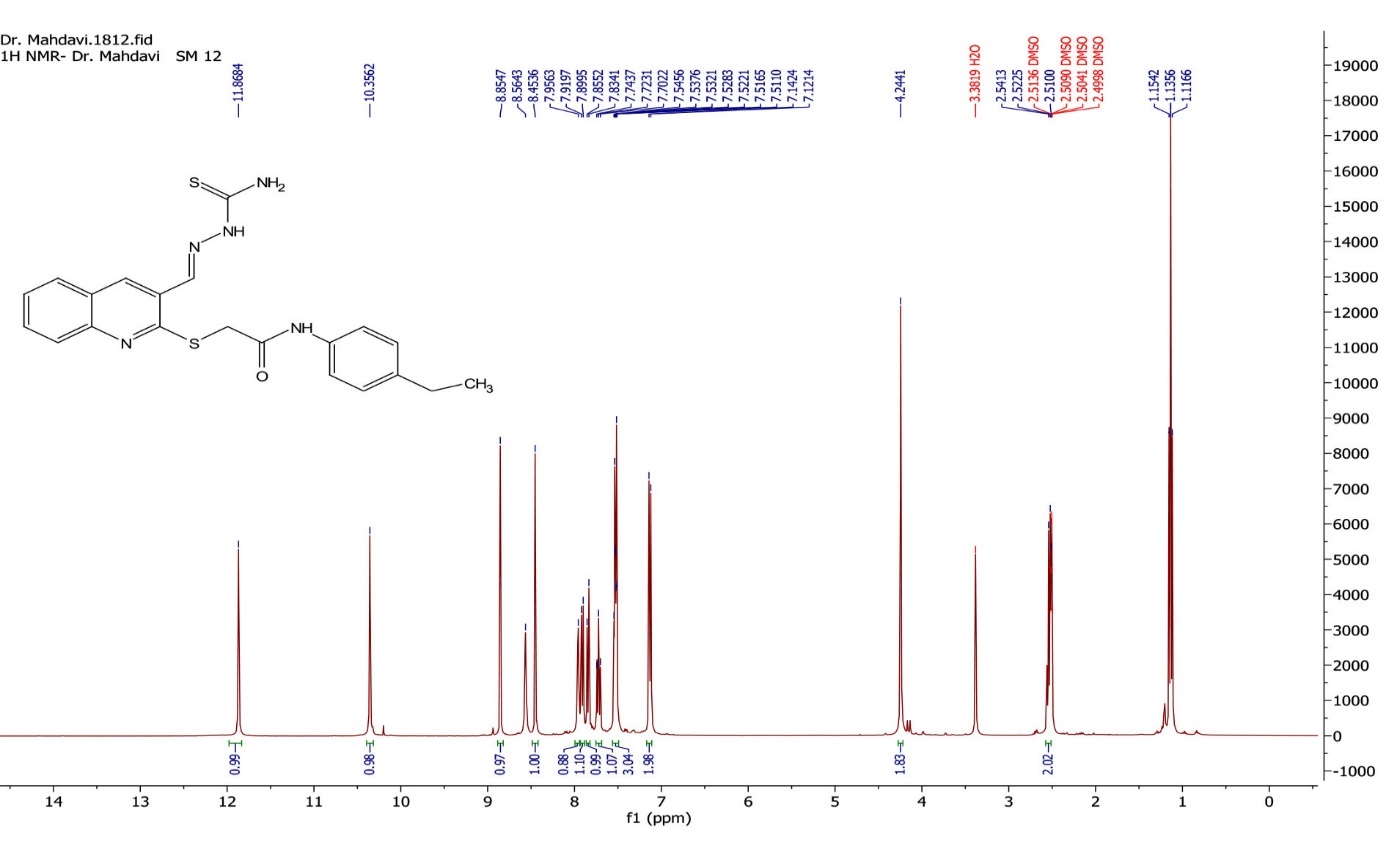
*

*
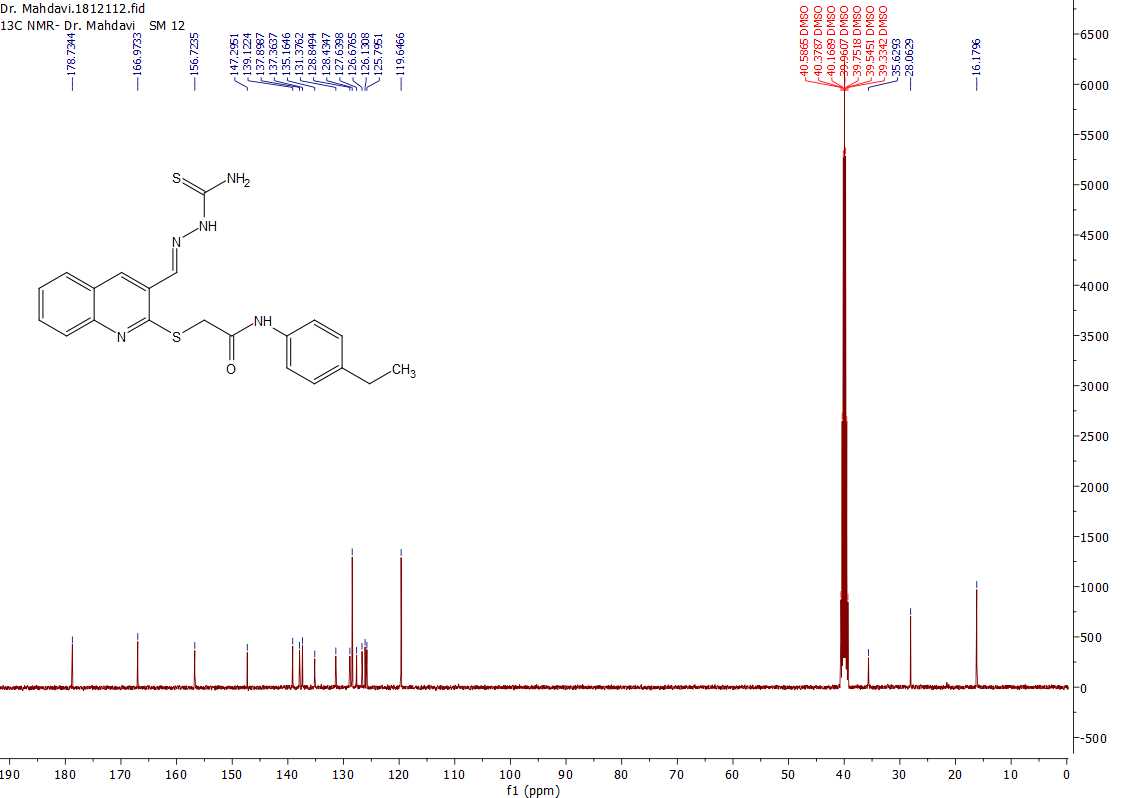
*

*Fig S4. (E)-2-((3-((2-carbamothioylhydrazono)methyl)quinolin-2-yl)thio)-N-(2,3-dimethylphenyl)acetamide (10d)*

*
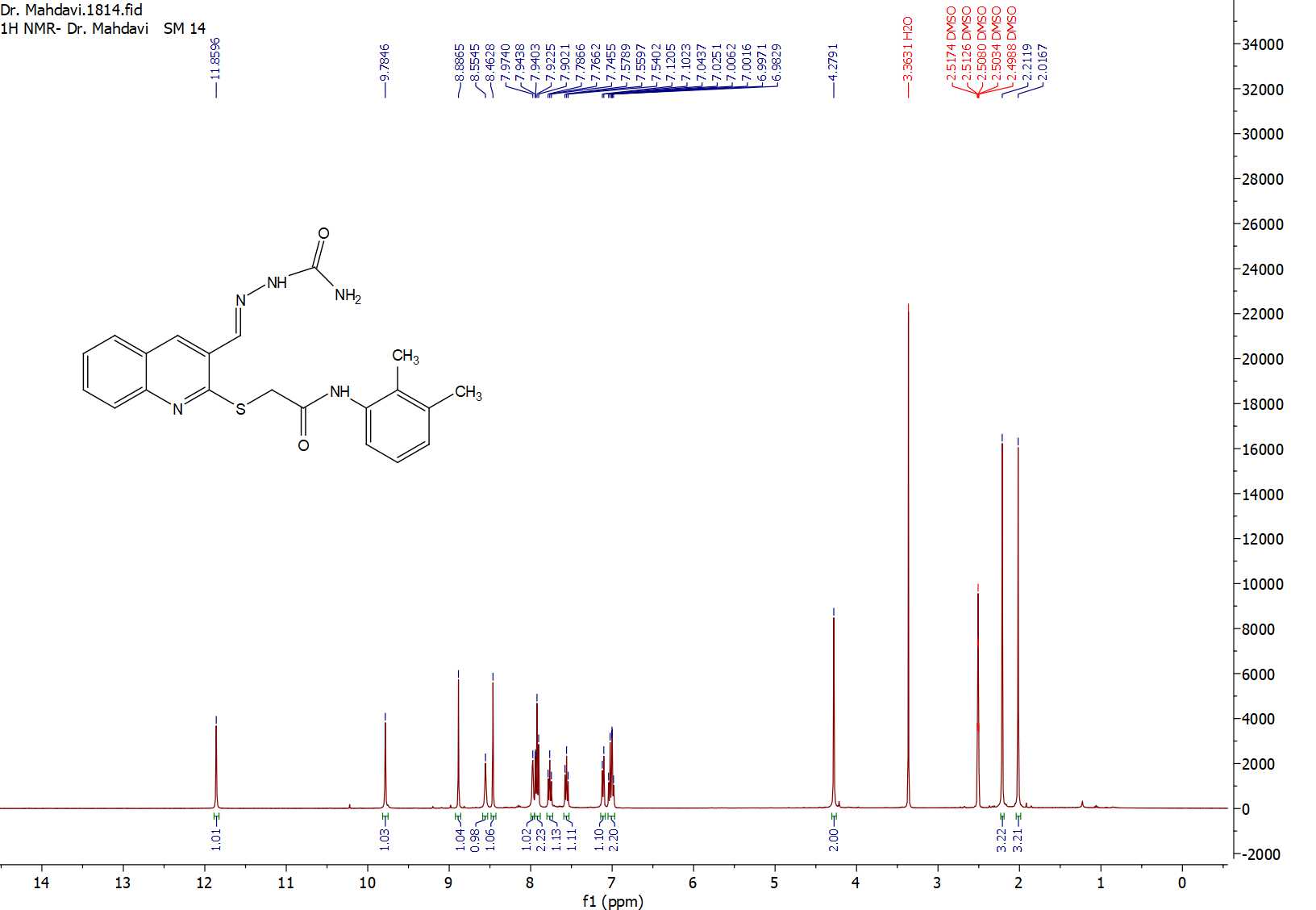
*

*
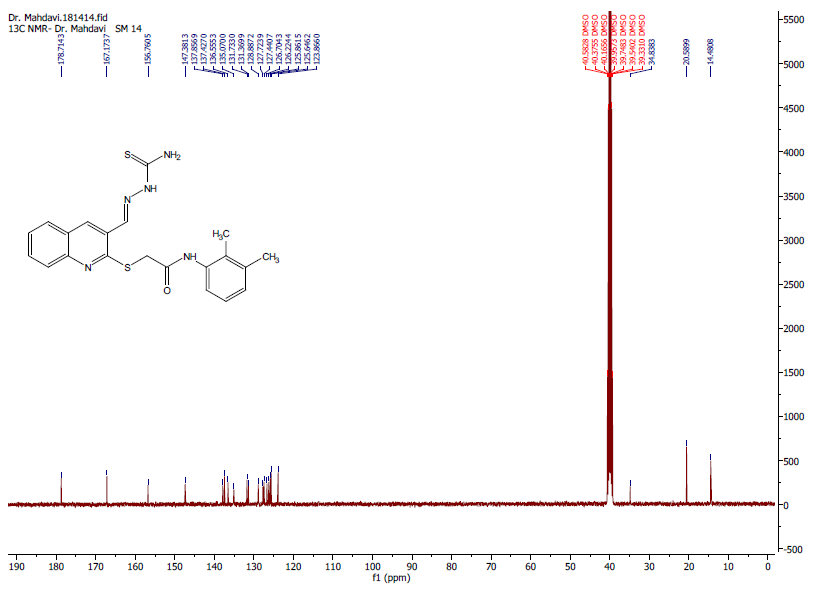
*

*Fig S5. (E)-2-((3-((2-carbamothioylhydrazineylidene)methyl)quinolin-2-yl)thio)-N-(4-methoxyphenyl)acetamide (10e)*

*
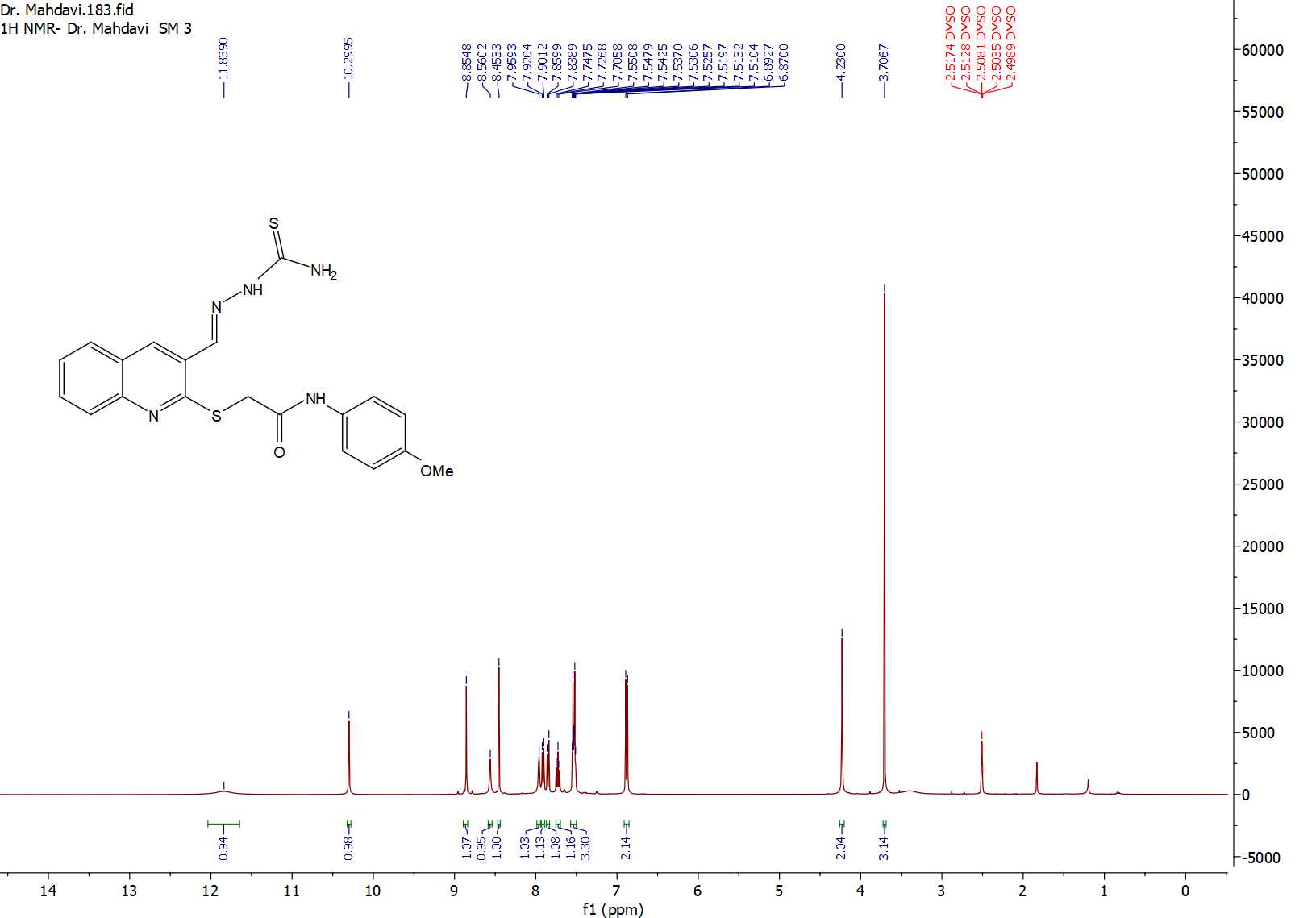
*

*
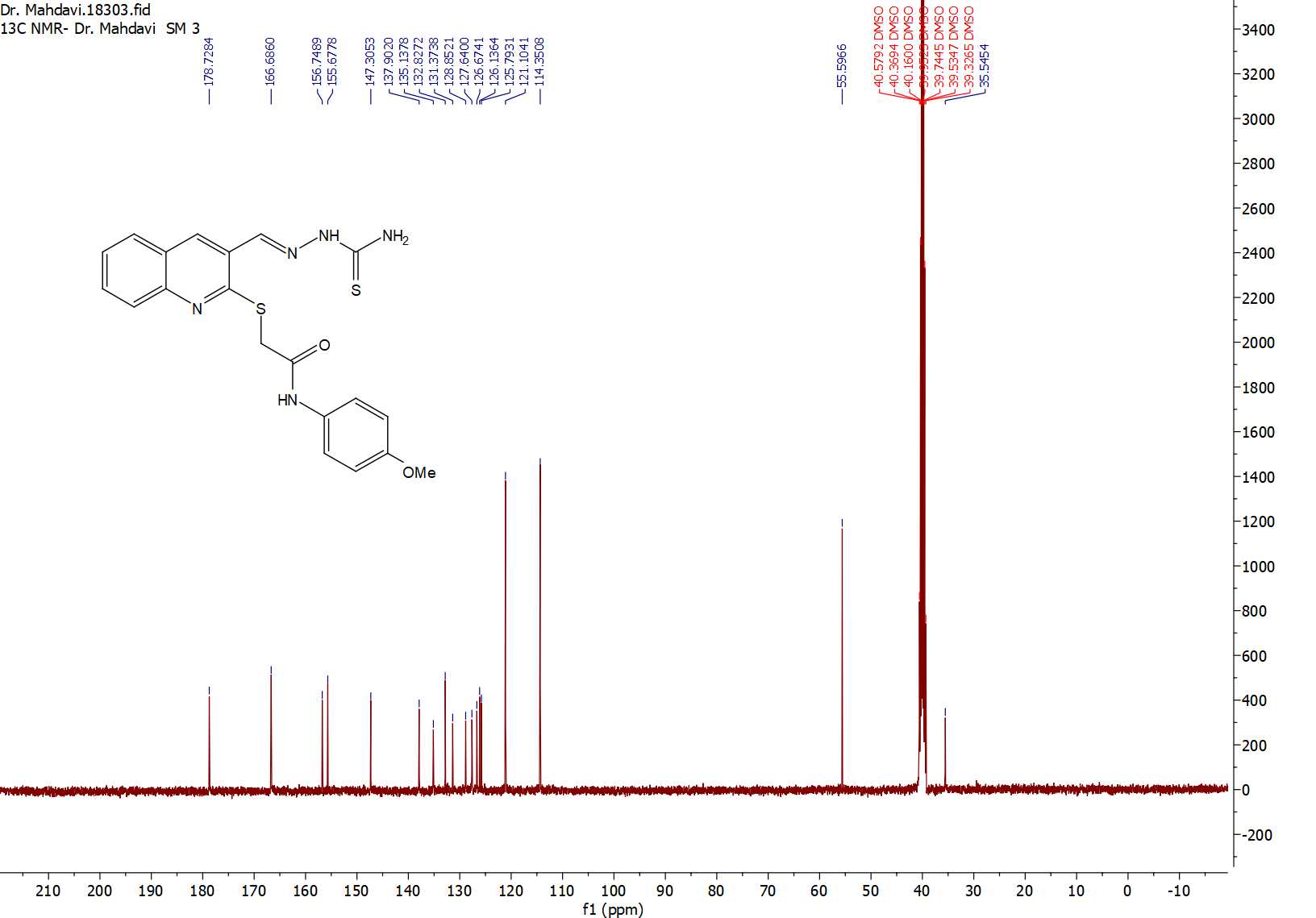
*

*Fig S6. (E)-2-((3-((2-carbamothioylhydrazineylidene)methyl)quinolin-2-yl)thio)-N-(4-nitrophenyl)acetamide (10f)*

*
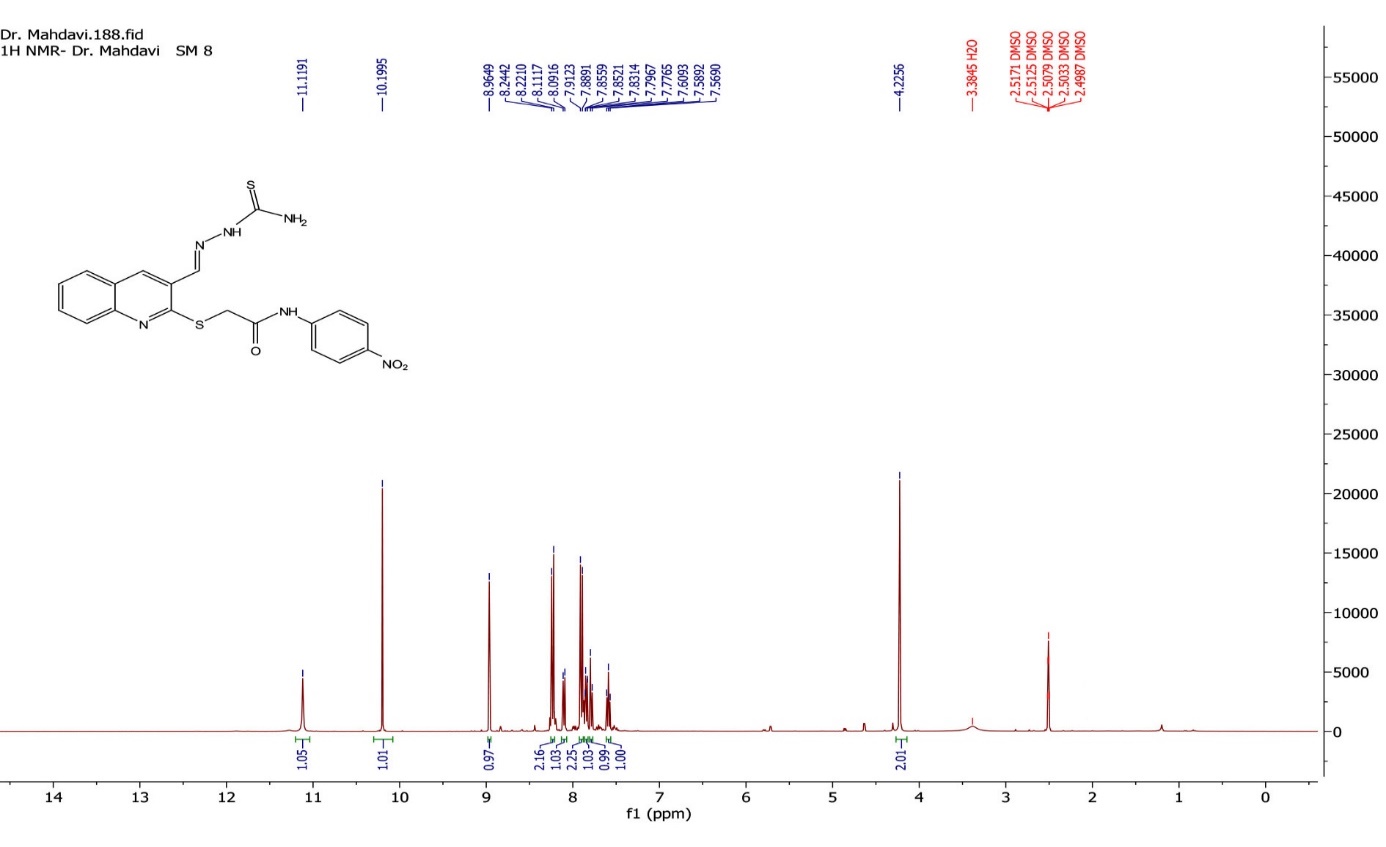
*

*
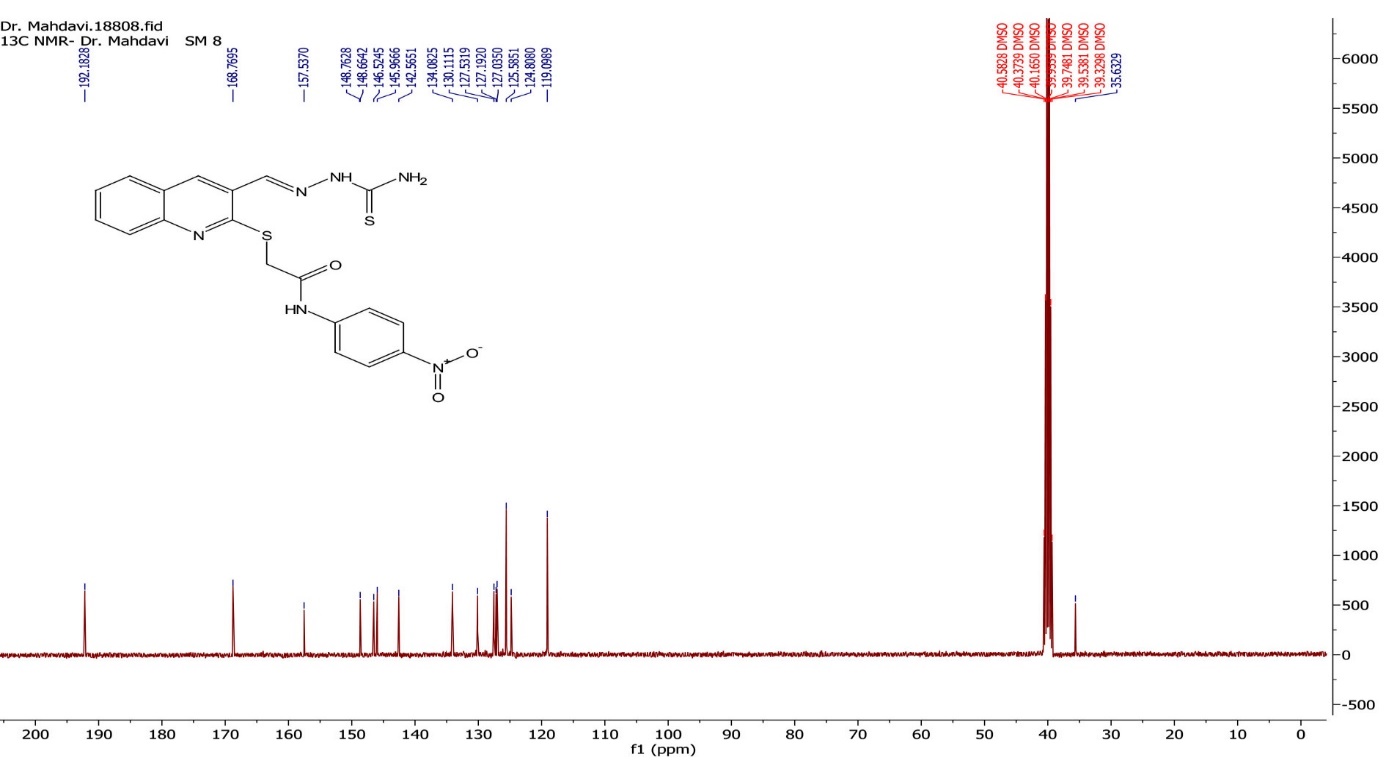
*

*Fig S7. (E)-2-((3-((2-carbamothioylhydrazono)methyl)quinolin-2-yl)thio)-N-(4-chlorophenyl)acetamide (10g)*

*
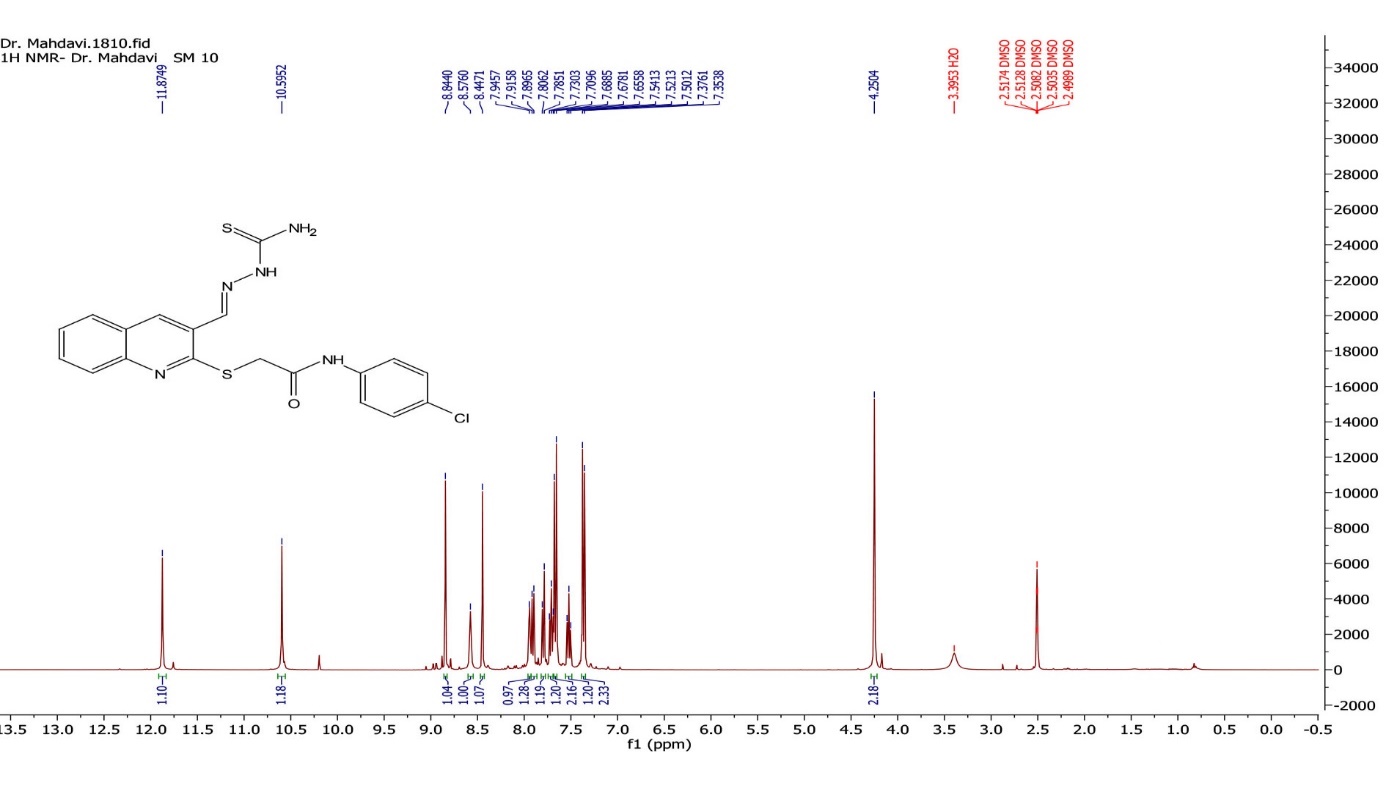
*

*
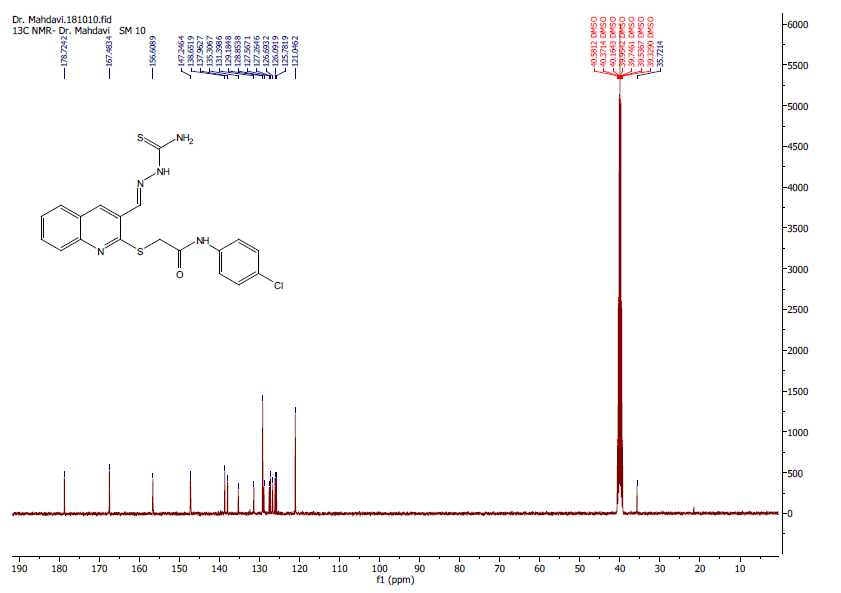
*

*Fig S8. (E)-2-((3-((2-carbamothioylhydrazono)methyl)quinolin-2-yl)thio)-N-(2,4-dichlorophenyl)acetamide (10h)*

*
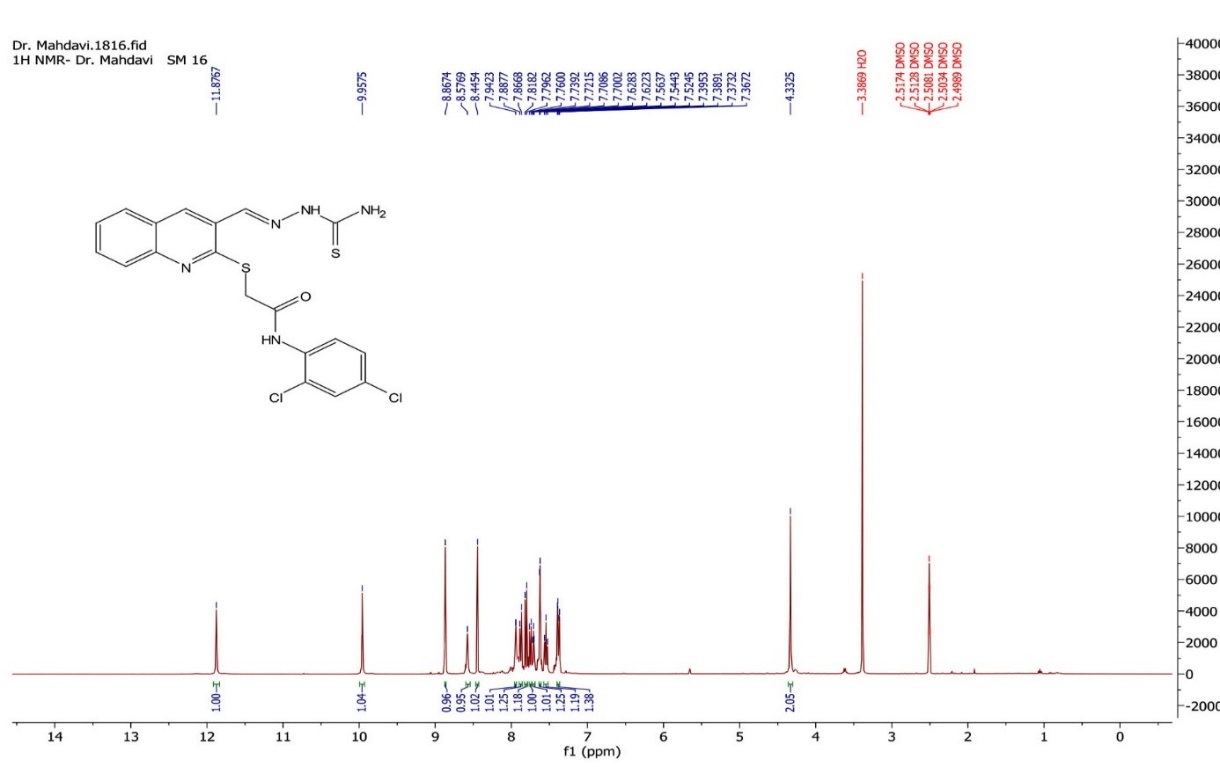
*

*
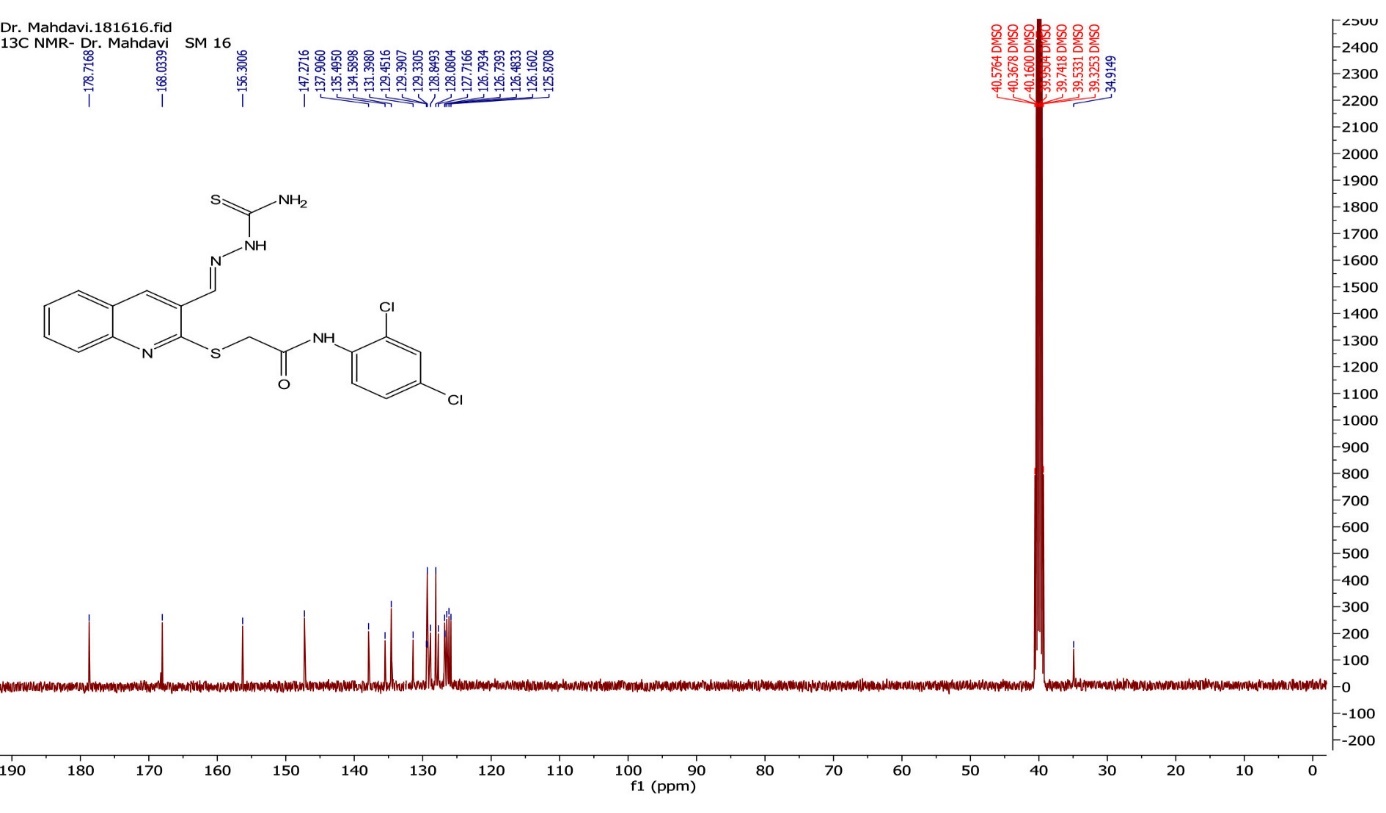
*

*Fig. S9 (E)-2-((3-((2-carbamothioylhydrazono)methyl)quinolin-2-yl)thio)-N-(4-fluorobenzyl)acetamide (10i)
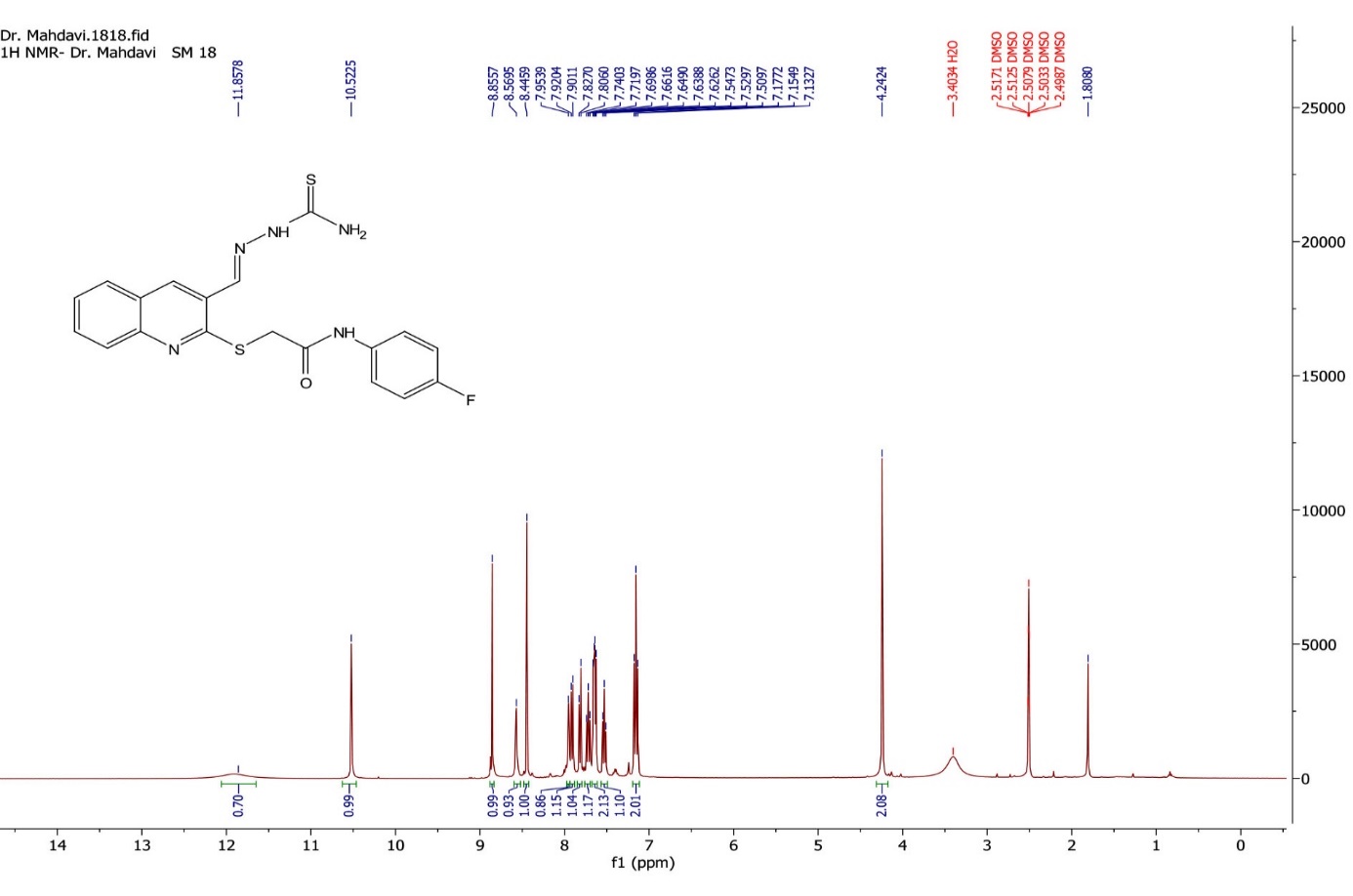
*

*
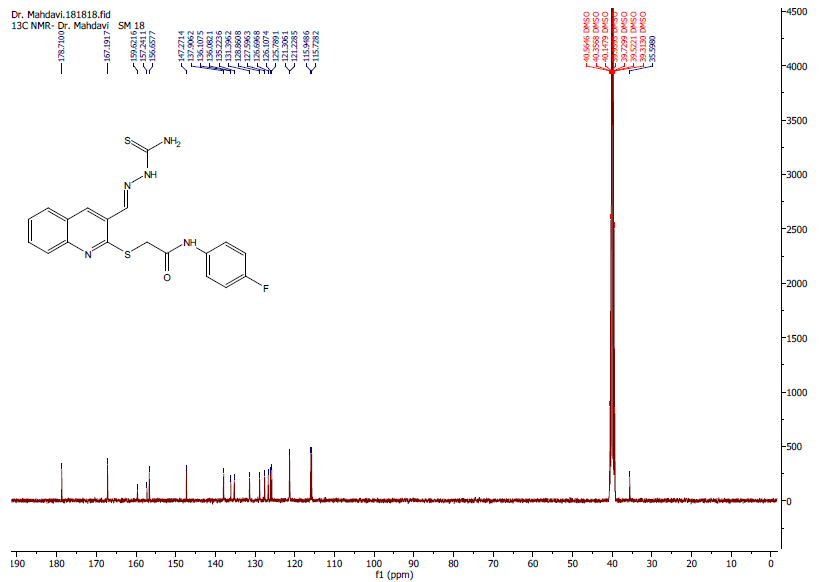
*

*Fig. S10. (E)-N-(2-bromophenyl)-2-((3-((2-carbamothioylhydrazono)methyl)quinolin-2-yl)thio)acetamide (10j)*

*
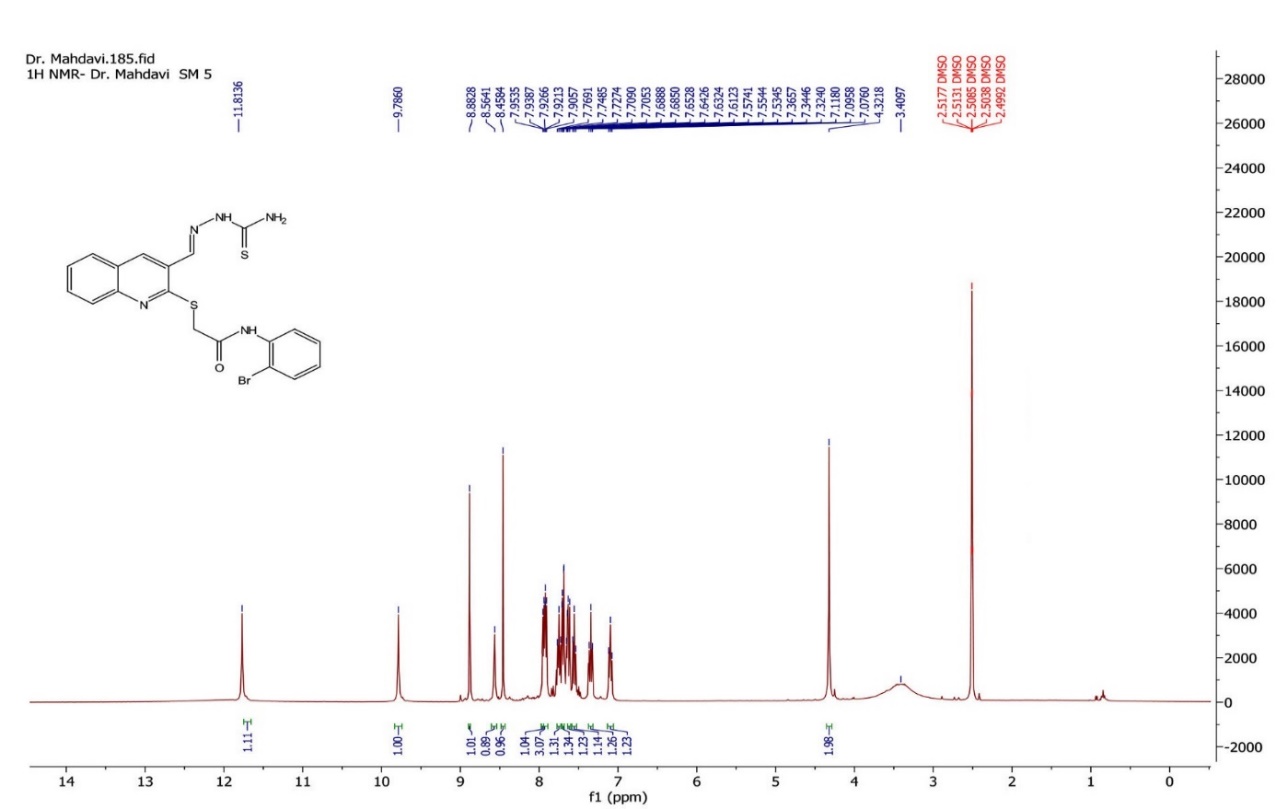
*

*
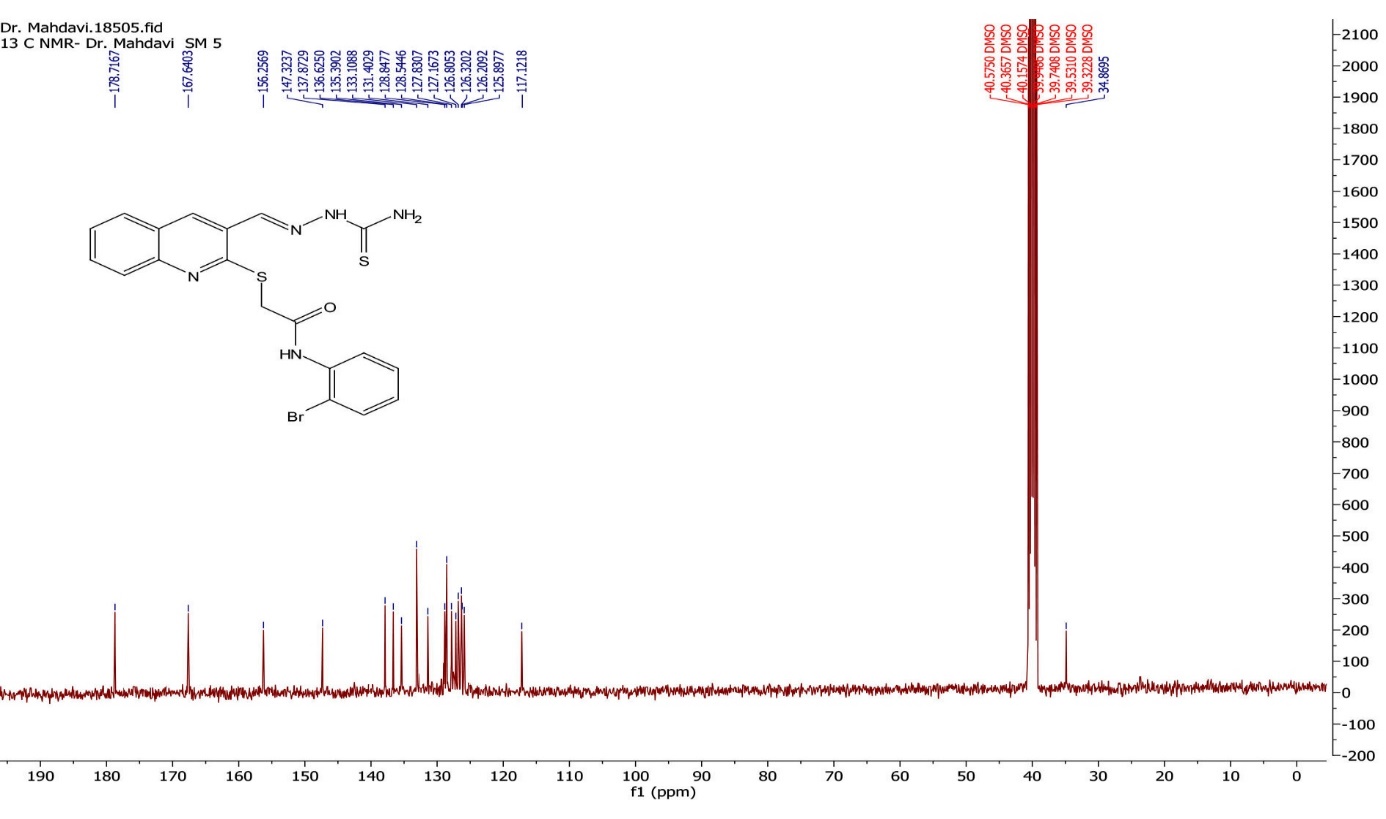
*

*Fig. S11. (E)-N-benzyl-2-((3-((2-carbamothioylhydrazono)methyl)quinolin-2-yl)thio)acetamide (10k)*

*
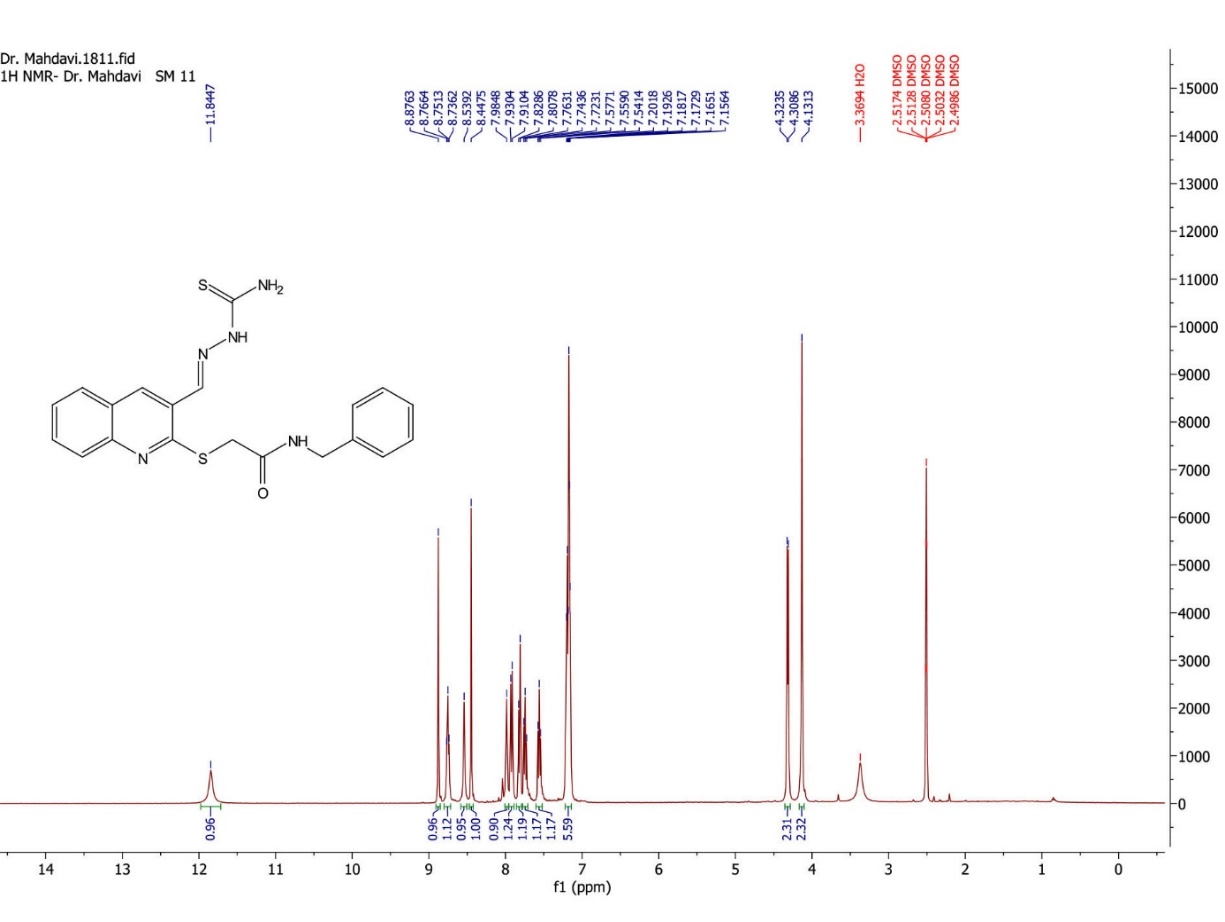
*

*
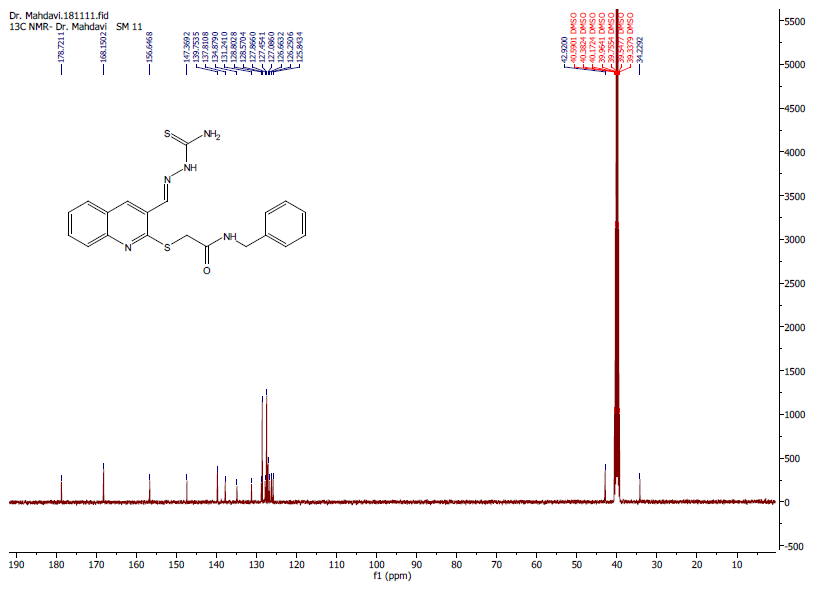
*

*Fig. S12. (E)-2-((3-((2-carbamothioylhydrazineylidene)methyl)quinolin-2-yl)thio)-N-(4-methylbenzyl)acetamide (10l)*

*
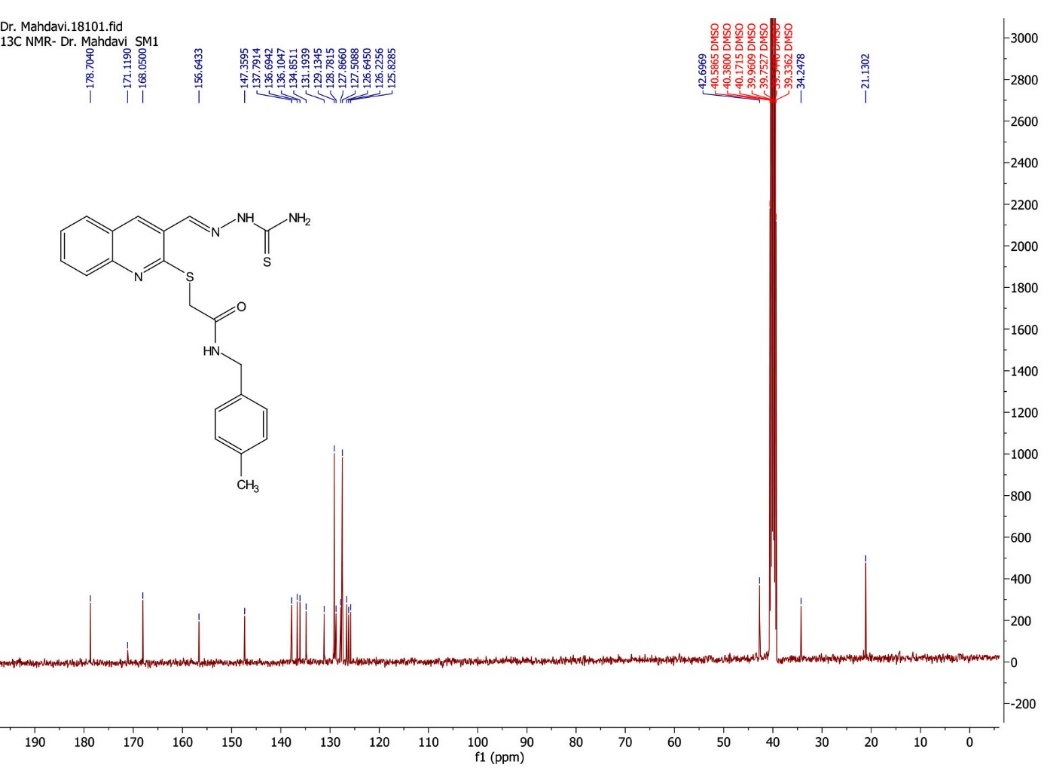
*

*
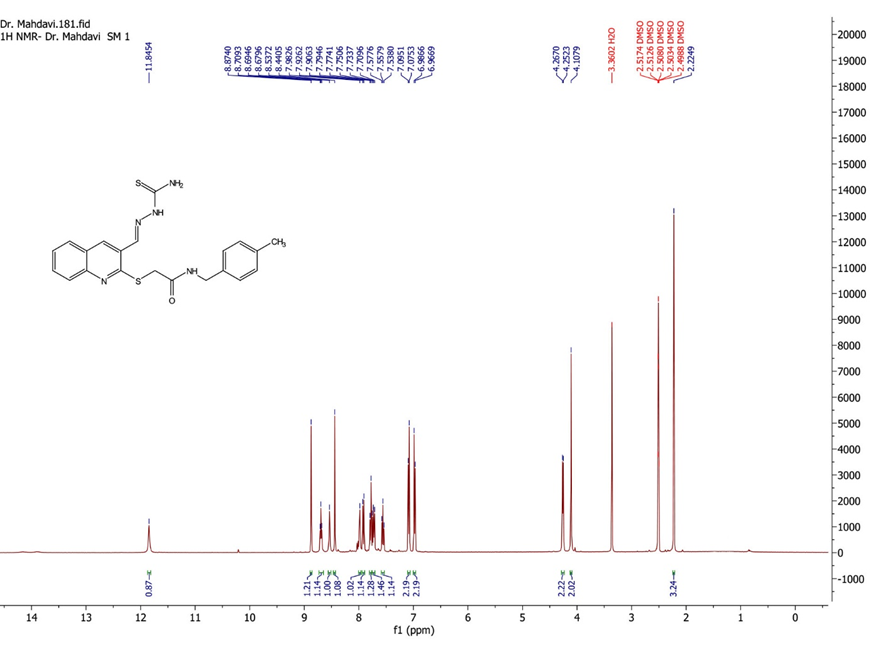
*

*Fig. S13. (E)-2-((3-((2-carbamothioylhydrazono)methyl)quinolin-2-yl)thio)-N-(4-fluorobenzyl)acetamide (10m)*

*
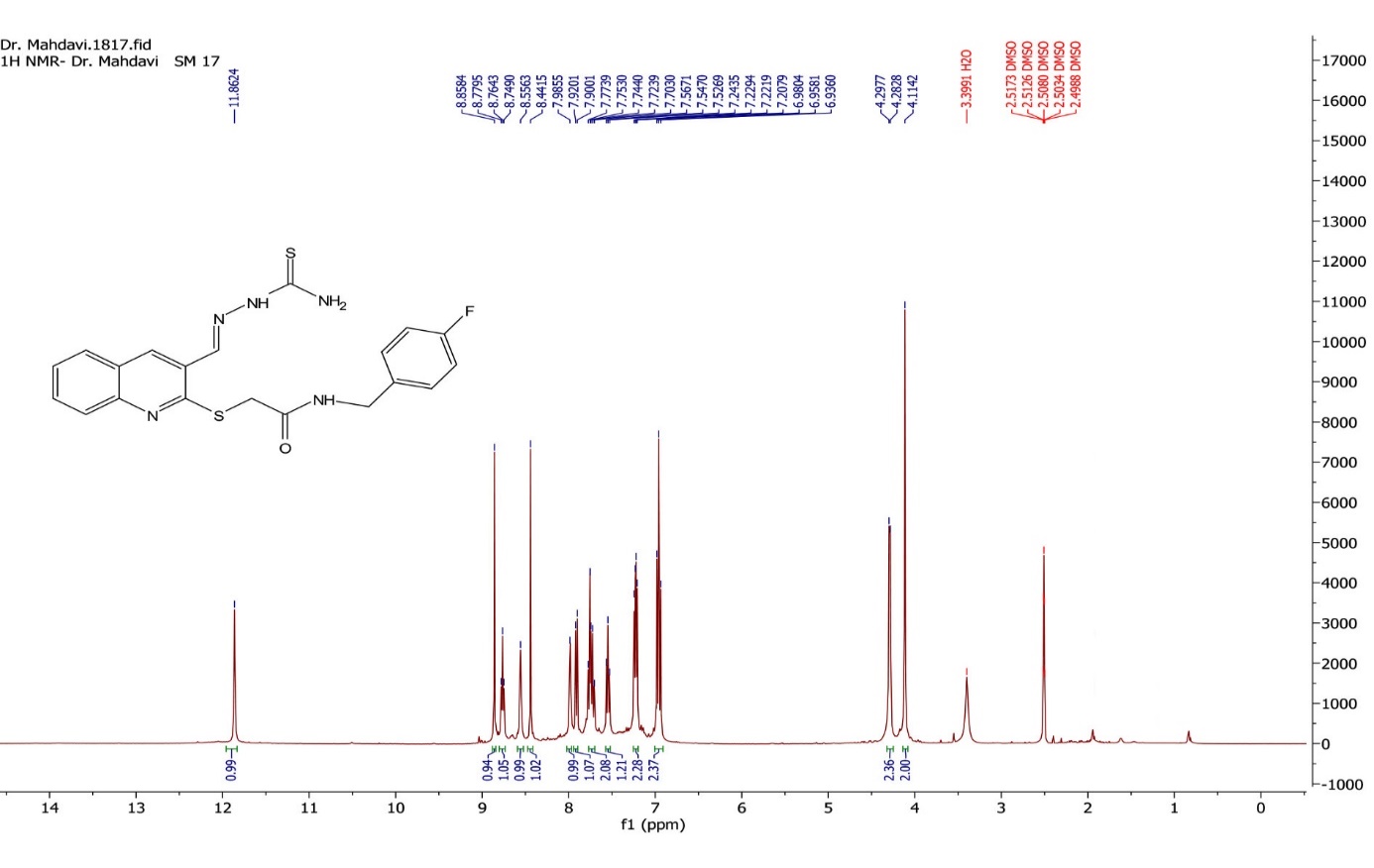
*

*
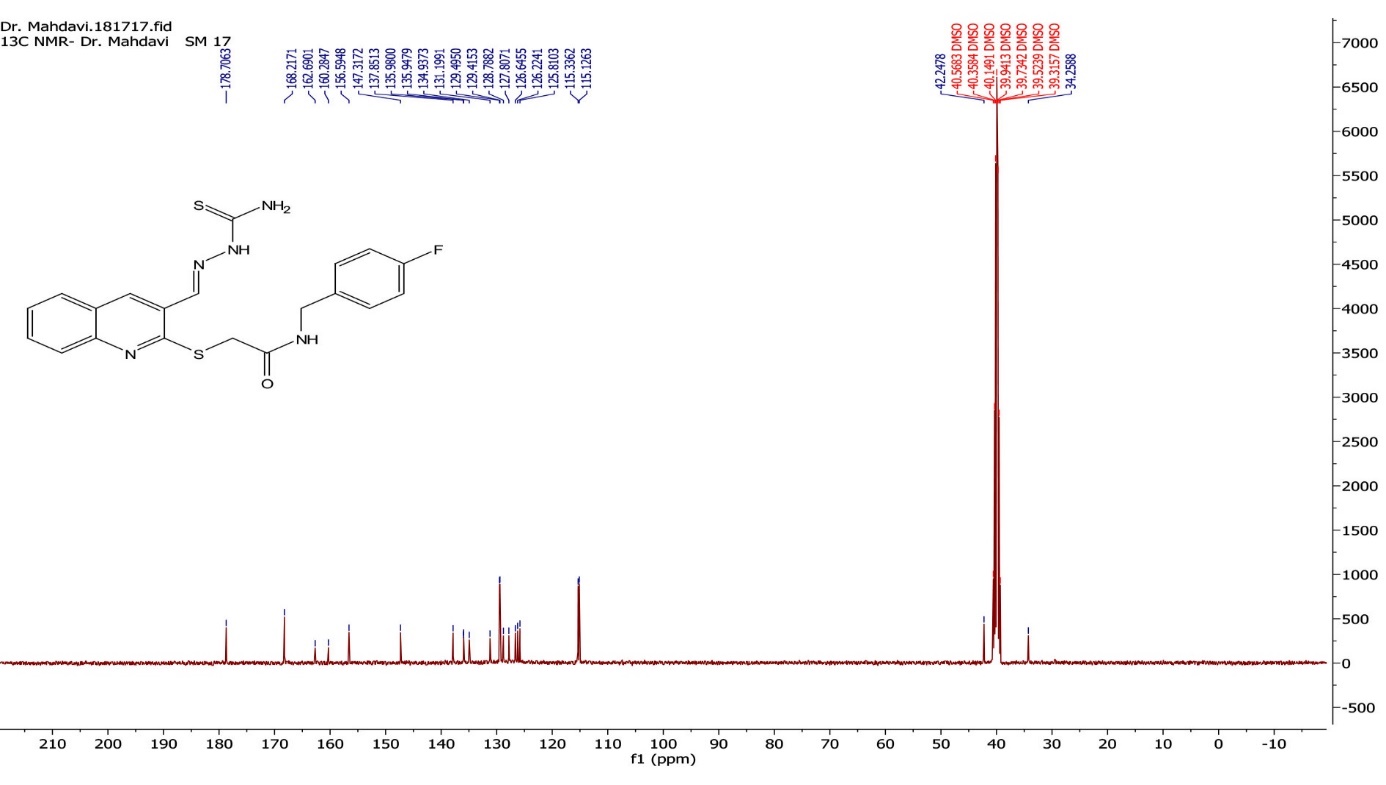
*
